# Association of food insecurity on gut microbiome and metabolome profiles in a diverse college-based sample

**DOI:** 10.1038/s41598-022-18515-y

**Published:** 2022-08-23

**Authors:** Alex E. Mohr, Paniz Jasbi, Kiley B. Vander Wyst, Irene van Woerden, Xiaojian Shi, Haiwei Gu, Corrie M. Whisner, Meg Bruening

**Affiliations:** 1 College of Health Solutions, Arizona State University, Phoenix, AZ USA; 2 School of Molecular Sciences, Arizona State University, Tempe, AZ USA; 3 College of Graduate Studies, Midwestern University, Glendale, AZ USA; 4 Community and Public Health, Idaho State University, Pocatello, ID USA; 5 Systems Biology Institute, Yale University, West Haven, CT USA; 6 Center for Translational Science, Florida International University, Port St. Lucie, FL USA; 7 Biodesign Institute Health Through Microbiomes Center, Arizona State University, Tempe, AZ USA; 8 Department of Nutritional Sciences, Pennsylvania State University, University Park, PA USA

**Keywords:** Nutrition, Metabolomics, Microbial communities

## Abstract

Voluntary caloric restriction (e.g., eating disorders) often results in alterations in the gut microbiota composition and function. However, these findings may not translate to food insecurity, where an individual experiences inconsistent access to healthy food options. In this study we compared the fecal microbiome and metabolome of racially and ethnically diverse first year college students (*n* = 60) experiencing different levels of food access. Students were dichotomized into food secure (FS) and food insecure (FI) groups using a validated, 2-question screener assessing food security status over the previous 30 days. Fecal samples were collected up to 5 days post survey-completion. Gut microbiome and metabolome were established using 16S rRNA amplicon sequencing, targeted liquid chromatography-tandem mass spectrometry, and gas chromatography-mass spectrometry. FI students experienced significantly greater microbial diversity with increased abundance of *Enterobacteriaceae* and *Eisenbergiella,* while FS students had greater abundance of *Megasphaera* and *Holdemanella*. Metabolites related to energy transfer and gut–brain-axis communication (picolinic acid, phosphocreatine, 2-pyrrolidinone) were elevated in FI students (*q* < 0.05). These findings suggest that food insecurity is associated with differential gut microbial and metabolite composition for which the future implications are unknown. Further work is needed to elucidate the longitudinal metabolic effects of food insecurity and how gut microbes influence metabolic outcomes.

## Introduction

Food insecurity, a socio-cultural construct tied to the lack of consistent access to healthy food, is a persistent public health problem that affects populations across the lifespan^1^. Food insecurity is related to poor dietary quality^2^ and increased risk for chronic diseases such as diabetes and cardiovascular disease^3^. College often marks an important transitory period where emerging adults gain independence not only in living situations and economic factors but also health behaviors including diet, physical activity, and sleep^4^. This, along with increased college access among low-income, underrepresented populations, may explain why 35–42% of college students appear to be at greater risk for food insecurity^5^. While more work is required to accurately capture food insecurity among college students^6^, food insecurity prevalence among college students is consistently reported to be 3–5 times higher than the national average^5^. Among college students, food insecurity has shown a persistent association with poorer physical, mental, and academic health outcomes^5^. Not surprisingly, dietary quality^7^ and food access^8^ (i.e., unused meal plans and type of meal plans) are lower, while anxiety and depression are higher among food insecure (FI) college students^9^. Importantly, recent research has documented that food insecurity is negatively associated with both short- and long-term academic outcomes^10,11^.

As a community of microbes inhabiting the gastrointestinal tract, the gut microbiota (GM) plays a critical role in development of the endocrine, immune, and central nervous systems^12^. Perturbations in this community are associated with a wide range of health issues including metabolic diseases (e.g., obesity, diabetes)^13^, gastrointestinal diseases (e.g., irritable bowel syndrome)^14^, and, more recently elucidated, neuropsychiatric disorders (e.g., depression, anxiety)^15^. In relation, the GM functionally compliments host metabolism, producing and modifying an array of metabolites from dietary, host, and biota-derived substrates^16^. Indeed, it is estimated that approximately half of the metabolites found in feces are produced/modified by the GM^17^. Despite the wealth of literature demonstrating GM as an indicator of health status, there is a paucity of studies exploring these relationships among college-aged adults^18,19^. Among college populations, unique microbial profiles have been found between weight-related changes^19^, BMI groups^19^, physical activity^18,20^, diet^18^, and screen time^18^. However, to our knowledge, no studies have evaluated differences in GM composition and the fecal metabolome among college students experiencing food insecurity.

To better understand the long-term health consequences of food insecurity among this population, exploration of the molecular and metabolic etiology is warranted. Food insecurity is theoretically associated with changes in the GM as low microbial diversity and an imbalance in pathogenic (e.g., *Staphylococcus*) and beneficial (e.g., *Bifidobacteria*) microbes are characteristic features of a calorie-dense, nutrient-poor diet which often associates with food insecurity^21,22^. Indeed, food insecurity may disrupt the GM composition and function leading to deficits in growth and development that contribute to disease risk^12^. To date, only one study has examined the relationship between food insecurity and the GM. Specifically, the abundance of *Veillonella* spp. was decreased among infants born to a small cohort of pregnant women who experienced food insecurity following Hurricane Maria in Puerto Rico (2017) as compared to infants born to food secure (FS) mothers^23^. Although, a valid measure of food insecurity was not used in this study, the prevalence of food insecurity was estimated to be 30%^23^. Other research has examined alterations in the GM in relation to nutritional outcomes that could coincide with food insecurity including malnutrition^22^, nutrient/food deprivation^24^, caloric deprivation by choice (anorexia^25^ and/or weight loss^26^), and intermittent fasting^27^. Therefore, it is essential to understand how the GM may shift under FI conditions, as little work has been done exploring this relationship.

While the literature on nutrient/food deprivation provides important insights, food insecurity differs from acute hunger (i.e., skipping a meal) or disordered eating, although sometimes disordered eating and food insecurity co-occur^28^. The psychological mechanisms of worrying (e.g., anxiety, depression) about access to food as opposed to choosing not to eat food are distinct. More research is critically needed to assess the relationship between food insecurity and the GM. Given this, we hypothesized that FI college students would have key microbial and metabolomic features distinct from FS college students.

## Results

### Study design and participant characteristics

**Figure 1:**
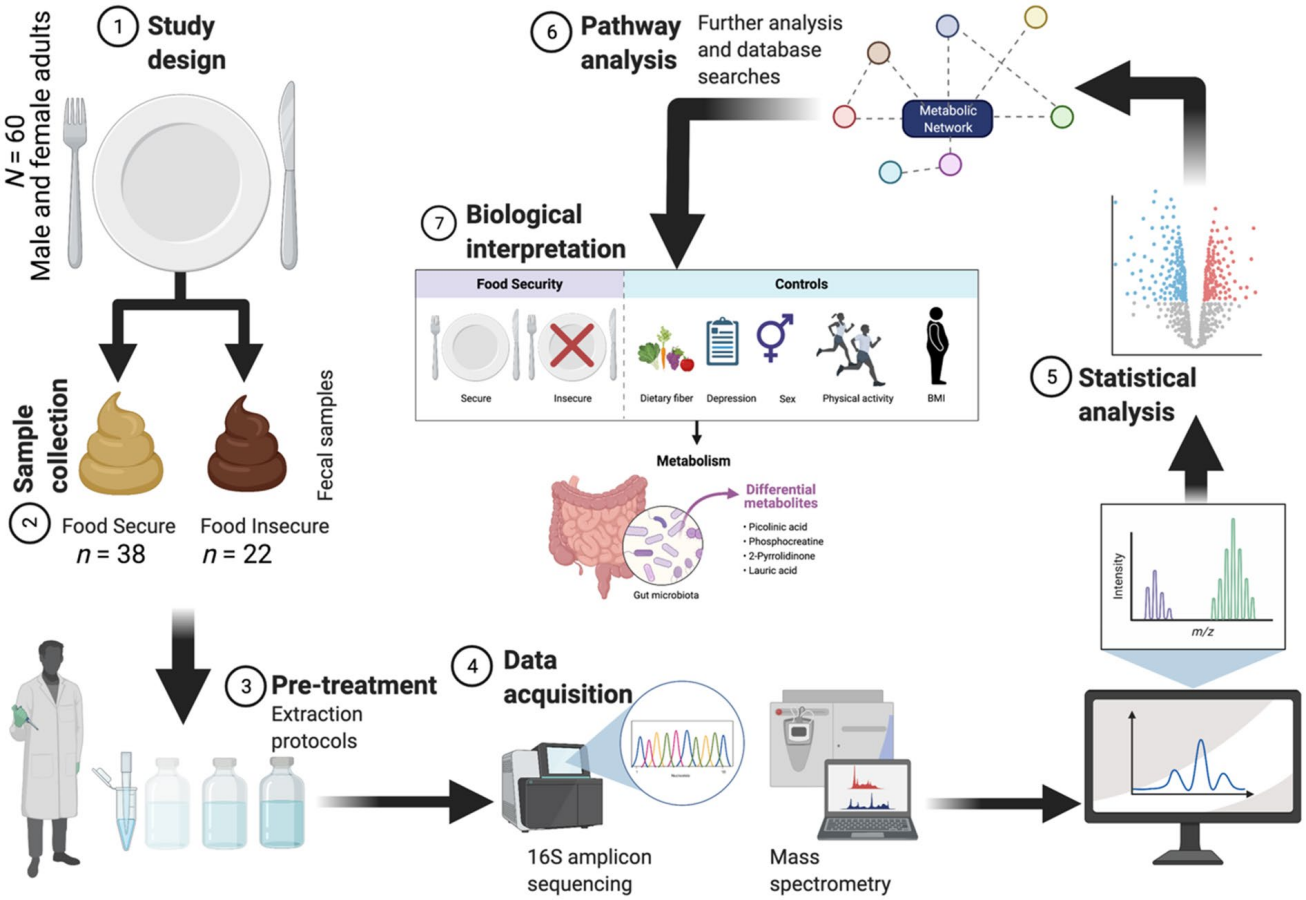
Overview of analytical workflow: male (*n* = 20) and female (*n* = 40) college students (mean age = 18.5 ± 0.7 years) were recruited and classified by food security status within the last 30 days. Fecal samples were collected and microbiome and metabolomic analyses were performed via 16S amplicon sequencing and mass spectrometry, respectively. Following data integration and metagenome assembly, statistical and predictive analyses were performed to articulate molecular signatures associated with recent food security. Created with BioRender.com.

Data assessed in this investigation were collected at a single time point as part of a feasibility study, representing a cross-sectional study of fecal microbiome and metabolomic data in a cohort of diverse college students; an overview of the analytical workflow is provided in Fig. 1. In brief, 60 participants each provided a fecal sample, demographic information, food security status, dietary recall, and moderate and vigorous physical activity (MVPA) data. Based on the food security status within the last 30 days, 38 individuals were classified as FS and 22 as FI (Table 1). Broadly assessing participant characteristics by food security classification, no significant differences were noted in constitutional factors such as age, sex, body mass index (BMI), and self-reported MVPA (*t* tests, *p* ≥ 0.115). As a proxy for economic condition, we did not detect a significant difference between classifications for Pell grant status (Fisher’s exact test, *p* = 0.791). Similarly, self-reported stress and depression did not differ between FS and FI participants, though depression was significantly correlated with MVPA (Spearman’s *ρ* = − 0.277, *p* = 0.032). In relation to diet, the overall mean percentage of kilocalories from carbohydrate, protein, and fat were 46.0 ± 17.9%, 16.9 ± 9.3%, and 37.1 ± 14.7%, respectively, with no significant differences by food security status. Notably, the mean daily consumption of dietary fiber for males (*n* = 20) and females (*n* = 40) was 12.21 ± 5.36 g/day and 12.70 ± 5.64 g/day, respectively, which fell below the recommended Adequate Intake for both males (38 g/day) and females (25–26 g/day). Moreover, mean dietary fat consumption was modestly elevated and outside of the acceptable macronutrient distribution range (AMDR) of 20–35%, whereas protein and carbohydrate consumption were within the AMDR of 10–35% and 45–65%, respectively. Finally, alcohol consumption, as quantified as the self-reported number of alcoholic beverages consumed over the previous 7 days, was not significantly different by food security status (Mann–Whitney *U* test, *p* = 0.278).

**Table 1:** Participant characteristics by food insecurity status.

	Total (*n* = 60)	Food insecure (*n* = 22)	Food secure (*n* = 38)
Age, mean ± SD	18.5 ± 0.7	18.4 ± 0.7	18.5 ± 0.7
**Sex, % (*n*)**			
Male	33.3 (*20*)	31.8 (*7*)	34.2 (*13*)
Female	66.7 (*40*)	68.2 (*15*)	65.8 (*25*)
**Race/ethnicity, % (*n*)**			
Non-Hispanic White	43.3 (*26*)	40.9 (*9*)	44.7 (*17*)
Non-Hispanic Black	8.3 (*5*)	22.7 (*5*)	0.0 (*0*)
Non-Hispanic Asian	15.0 (*9*)	4.5 (*1*)	21.1 (*8*)
Hispanic	25.0 (*15*)	13.6 (*3*)	31.6 (*12*)
Other	8.3 (*5*)	18.2 (*4*)	2.6 (*1*)
**Body Mass Index (kg/m**^**2**^**), mean ± SD**	24.4 ± 5.9	25.7 ± 7.3	23.7 ± 4.8
< 18.5 kg/m^2^% (*n*)	6.7 (*4*)	4.5 (*1*)	7.9 (*3*)
18.5–24.9 kg/m^2^% (*n*)	60.0 (*36*)	59.1 (*13*)	60.5 (*23*)
25.0–29.9 kg/m^2^% (*n*)	18.3 (*11*)	13.6 (*3*)	21.1 (*8*)
≥ 30.0 kg/m^2^% (*n*)	15.0 (*9*)	22.7 (*5*)	10.5 (*4*)
**Screen time (min/day)**	206.8 ± 116.6	231.8 ± 114.9	192.2 ± 116.7
Moderate-to-vigorous physical activity (min/day)	51.3 ± 30.9	46.7 ± 31.2	54.0 ± 30.9
Self-reported depression	2.2 ± 0.7	2.4 ± 0.8	2.1 ± 0.6
Pell grant recipient, % (*n*)	53.3 (32)	50.0 (11)	55.3 (21)
**Diet, mean ± SD**	1614 ± 589	1644 ± 638	1595 ± 566
Carbohydrates (g)	186.1 ± 72.5	184.7 ± 79.5	186.9 ± 69.0
Sugar (g)	75 ± 46.1	76.4 ± 54.9	74.3 ± 40.6
Fiber (g)	12.6 ± 5.5	11.9 ± 4.8	12.9 ± 5.9
Protein (g)	68.3 ± 37.7	71.6 ± 52.9	66.2 ± 24.8
Fat (g)	66.5 ± 26.5	70.4 ± 28.9	62.9 ± 24.9
**Alcohol intake (no. beverages over the last 7 days)**	3.6 ± 0.7	4.2 ± 1.1	3.3 ± 0.9

*SD* standard deviation.

### Selected diversity metrics of the fecal microbiome differed by food security status

**Figure 2:**
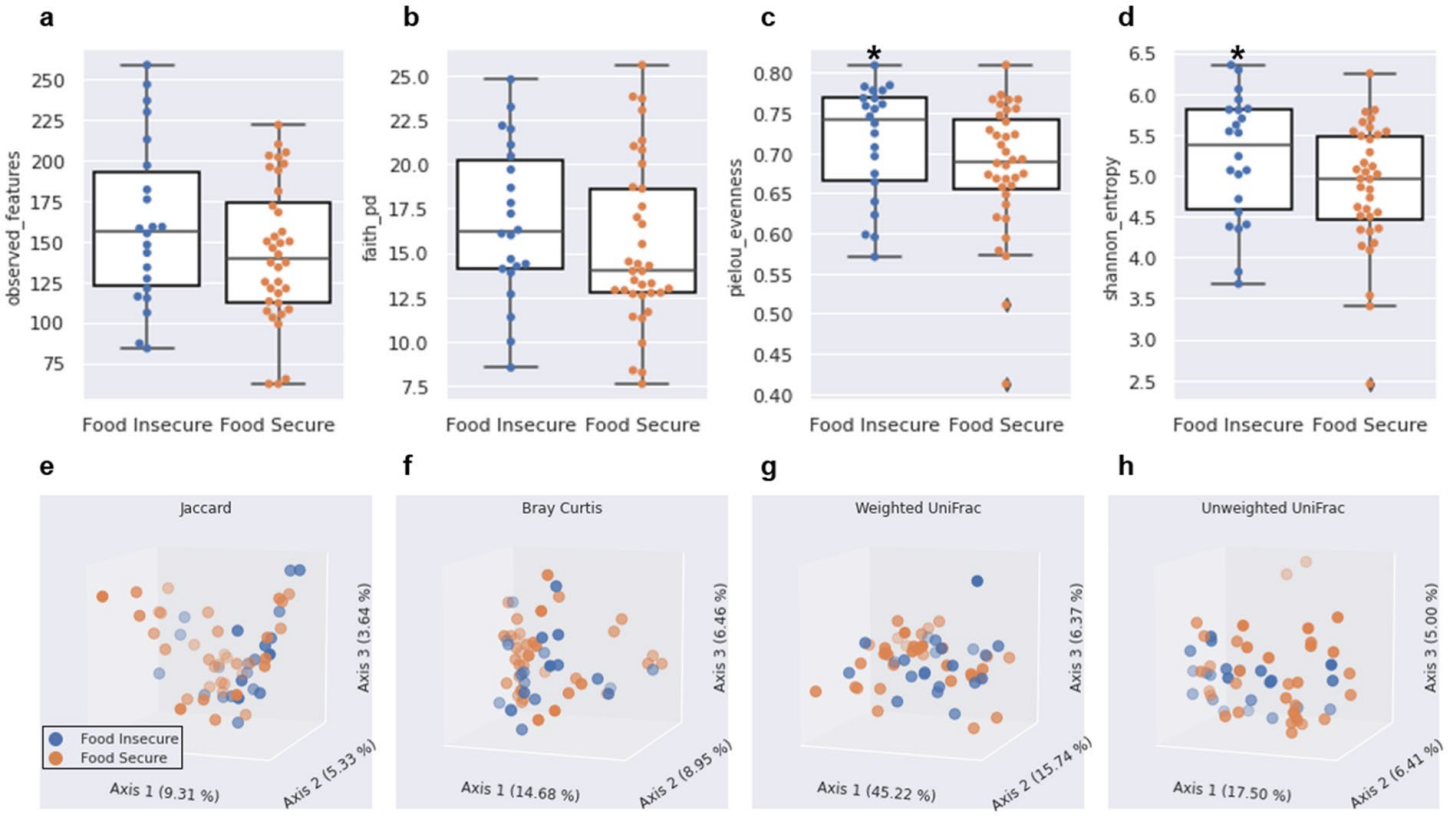
Alpha diversity boxplots showing; (**a**) Observed features, (**b**) Faith’s PD, (**c**) Pielou’s E, and (**d**) Shannon index, by food security status. Boxes denote the interquartile range (IQR) between the first and third quartiles, and the horizontal line defines the median. Whiskers represent the smallest (y-min) and largest (y-max) observations within 1.5 times the IQR from the first and third quartiles. Asterisk denotes significance at *p* < 0.05. Principal coordinates’ analysis for beta diversity metrics; (**e**) Jaccard, (**f**) Bray Curtis, (**g**) Weighted UniFrac, and (**h**) Unweighted UniFrac distances by food security status.

Controlling for the covariates, sex, BMI, fiber intake, and self-reported depression and MVPA, alpha diversity metrics, observed features and Faith’s PD were not significantly different by food security status (*p* ≥ 0.143; Fig. 2a,b, respectively). In comparison, individuals classified as FI had significantly greater alpha diversity values compared to FS individuals for Pielou’s evenness (*p* = 0.049; Fig. 2c) and Shannon index (*p* = 0.047; Fig. 2d). Using the same covariates for beta diversity analyses, only Jaccard was significant (*p* = 0.050; Fig. 2e), while Bray Curtis (Fig. 2f), Unweighted UniFrac (Fig. 2g), and Weighted UniFrac (Fig. 2h) were not (*p* ≥ 0.080) (Supplementary Table S1).

### Microbial differential abundance and correlations differs by food security status

**Figure 3:**
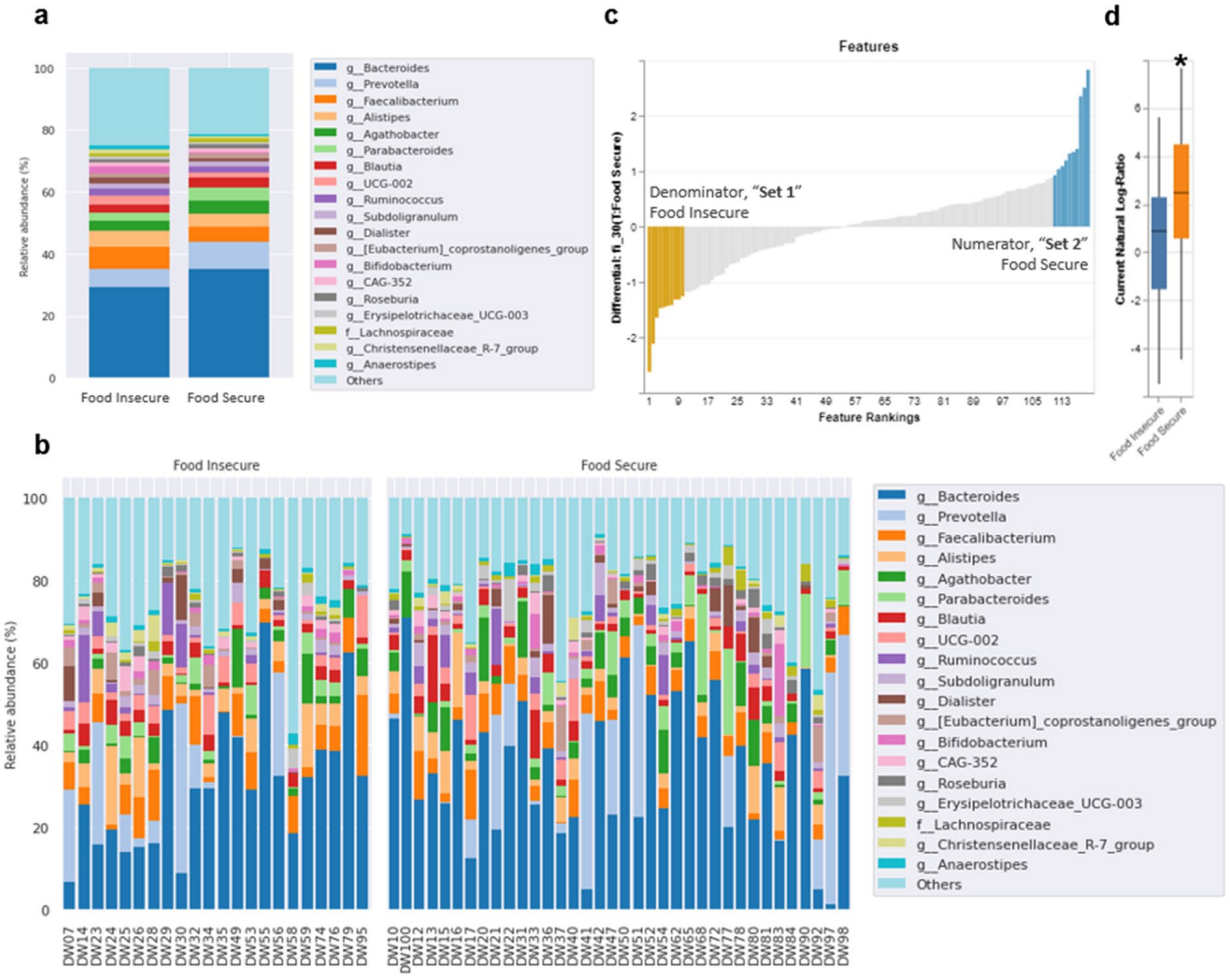
Taxonomic analysis of fecal microbiome by food security status. (**a**) Taxonomy bar plot of the top 20 most abundant taxa at the genus level for both groups (*n* = 60). Less abundant taxa are not displayed. (**b**) Taxonomy bar plot of individual subjects at the genus level by FI (*n* = 22) and FS status (*n* = 38). Less abundant taxa are not displayed. Note, where resolution at the genus level was not possible taxa are described at the lowest feature level obtained (i.e., f = family). (**c**) Comparison of the log ratio of the 10 lowest (“Set 1”) and 10 highest (“Set 2”) ranked taxa associated with food security status (after filtering, *n* = 58). (**d**) FS individuals had a significantly greater log ratio of set 2 compared to set 1 (Mann–Whitney *U* test: *p* < 0.05).

After quality control, taxonomic assignment mapped to the SILVA database (v. 138.1) identified 218 unique amplicon sequence variants (ASVs) (phylum: 14; class: 23; order: 47; family: 74; genus: 166; Fig. 3a,b). Differential abundance was assessed on filtered feature data by Songbird^29^, a compositionally aware multinomial regression method which provides rankings of taxa based on their logarithm of fold change (FC) between conditions known as “differentials”. As with the diversity analysis, the same covariates of sex, BMI, fiber intake, and self-reported depression and MVPA were entered into the model. The taxon differentials between the two groups produced by Songbird were then entered into Qurro^30^ to compute the log ratios of taxa of which we selected the 10 lowest (“Set 1”) and the 10 highest features (“Set 2”; Fig. 3c; Supplementary Table S2). Comparing the log ratio of these two sets, food security had a significantly greater log ratio of Set 2 compared to Set 1 (Mann–Whitney *U* test; *p* = 0.012, Cohen’s *d* = 0.726; Fig. 3d). Specifically, food security had a greater abundance of *Clostridia*, *Megasphaera*, and *Holdemanella* (FS:FI, log-FC > 2.0), whereas food insecurity had a greater abundance *Enterobacteriaceae* and *Eisenbergiella* (FI:FS, log-FC > 2.0).

**Figure 4:**
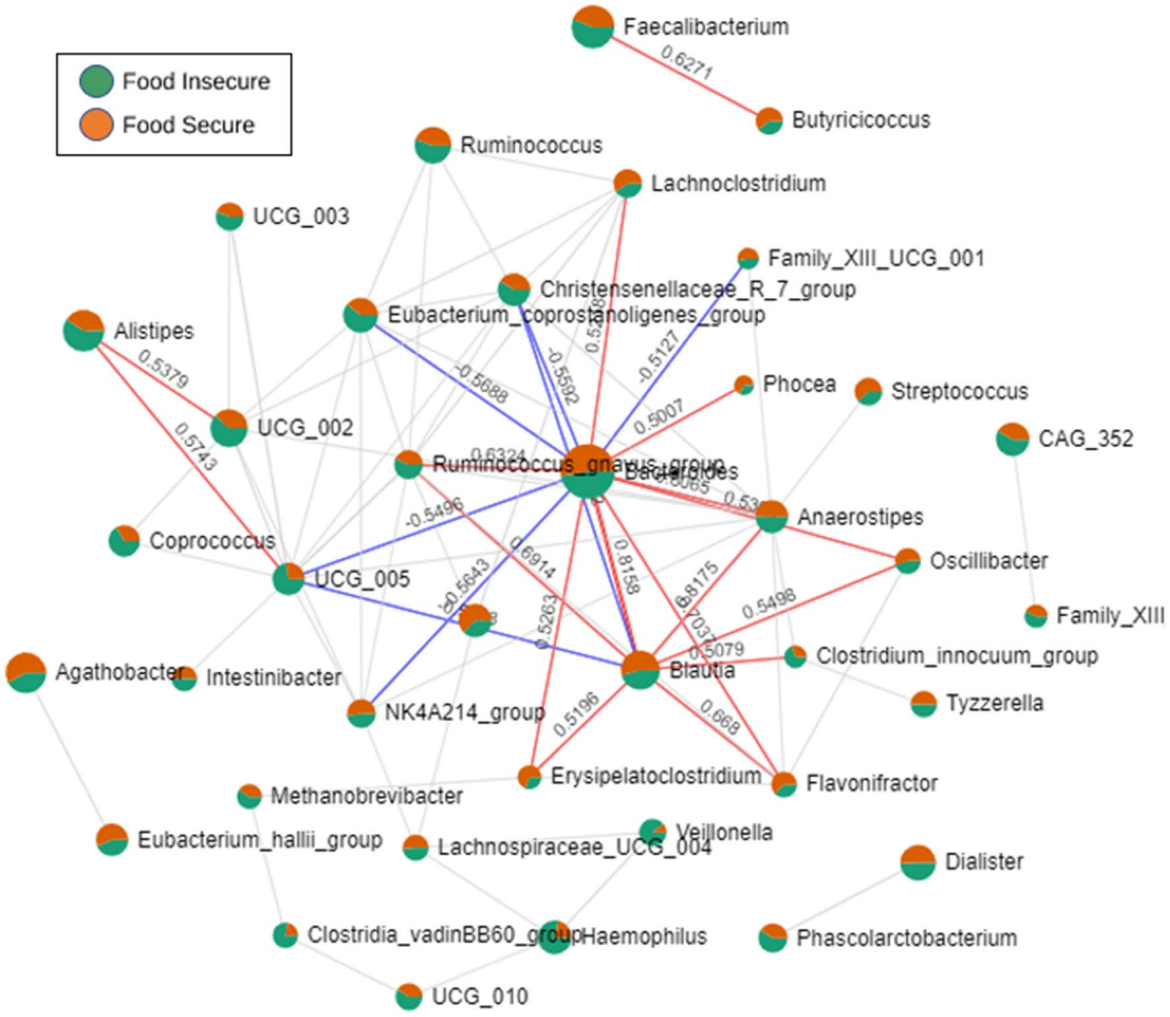
Correlation analysis of taxa at the genus level between food insecurity and food security status. Network analysis between abundant taxa constructed from SparCC correlation coefficients (|*R*^2^| > 0.5, *p* < 0.01). Note, each node represents a microbe by abundance between food security status, with the lines (and corresponding values) indicating the correlation coefficients between the genera. Blue edges are negative correlations and red edges mean positive correlations.

Next, to identify relationships between taxa by food security status, a correlation network was constructed using SparCC (via FastSpar) to account for compositional sparsity of microbiome data^31^. Based on the number of connections and the strength of associations (|*R*^2^| > 0.5, *p* < 0.01), *Bacteroides* (5 negative and 8 positive correlations), *Blautia* (2 negative and 7 positive correlations), *Alistipes* (2 positive correlations), and *Faecalibacterium* (1 positive correlation), dominated the network (Fig. 4). Upon further examination via pattern search analysis of each of these individual taxa, *Bacteroides* was significantly positively correlated to the genera, *Anaerostipes*, *Flavonifractor*, and *Ruminococcus* gnavus group (*R*^2^ > 0.6, *q* < 0.05); *Blautia* was significantly positively correlated to *Ruminococcus* gnavus group and *Flavonifractor* (*R*^2^ > 0.6, *q* < 0.05); *Alistipes* was significantly positively correlated to the genera, *UCG_005* and *UCG_002* (*R*^2^ > 0.6, *q* < 0.05); *Faecalibacterium* was significantly positively correlated to the genera, *Butyricicoccus* (*R*^2^ > 0.6, *q* < 0.05). These results are displayed in Supplementary Fig. S1.

### Influence of food security status on the predicted functional profile of the gut microbiome

The Phylogenetic Investigation of Communities by Reconstruction of Unobserved States 2 (PICRUSt 2) pipeline^32^ was used to infer the functional profile of the GM data. The Kyoto Encyclopedia of Genes and Genomes (KEGG) outputs were analyzed and illustrated with Songbird and Qurro, respectively. Accounting for the covariates of sex, BMI, fiber intake, and self-reported depression and MVPA, the log ratios of the 10 lowest (“Set 1”) and the 10 highest predicted features were selected (“Set 2”; Supplementary Fig. S2; Supplementary Table S3). Comparing the log ratio of these two sets, food security had a significantly greater log ratio of Set 2 compared to Set 1 (Mann–Whitney *U* test; *p* = 0.007, Cohen’s *d* = 0.756; Supplementary Fig. S2). Considering features with a log-FC > 2.0, food security was most strongly associated with UDP-N-acetylmuramoylpentapeptide-lysine N(6)-alanyltransferase (log-FC = 2.054), whereas food insecurity was more associated with adenosylcobinamide hydrolase (log-FC = 2.089).

Taxon set enrichment analysis (TSEA) using a least absolute shrinkage and selective operator (LASSO) was performed to assess predicted functional profiles of host-intrinsic factors such as disease as well as host-extrinsic factors such as diet and lifestyle using 239 and 118 taxon sets, respectively. TSEA revealed FI participants had significant increases in taxon sets related to anorexia (*p* = 0.011), urogenital schistosomiasis (*p* = 0.011), chronic heart failure (*p* = 0.024), and depression (*p* = 0.024). Conversely, FI subjects had significant decreases in taxa related Crohn’s disease (*p* = 0.001), type I diabetes (*p* = 0.003), having an overweight or obese mother (*p* = 0.003), myocardial infarction (*p* = 0.008), and resistance to immune checkpoint inhibitors (*p* = 0.036). TSEA also showed that FI individuals had significant decreases in taxon sets associated with the consumption of red wine (*p* = 0.007), fruits (*p* = 0.025), and coffee (*p* = 0.032). TSEA results are presented in Table 2.

**Table 2:** Results of taxon set enrichment analysis (TSEA) showing taxon set, total taxa in each set, taxa sequenced, and associated *p* values for the food insecurity group.

Taxon set*	Total	Hits	*p*
**Disease**			
Crohn’s disease (decrease)	21	4	0.001
Type I diabetes (decrease)	13	3	0.003
Overweight/obese mother (decrease)	4	2	0.003
Myocardial infarction (decrease)	6	2	0.008
Anorexia (increase)	7	2	0.011
Urogenital schistosomiasis (increase)	7	2	0.011
Chronic heart failure (increase)	1	1	0.024
Depression (increase)	1	1	0.024
Resistance to immune checkpoint inhibitors (decrease)	13	2	0.036
**Diet and lifestyle**			
Red wine (decrease)	5	3	0.007
Fruits (decrease)	3	2	0.025
Coffee (decrease)	8	3	0.032

*Taxon sets analyzed as FI/FS.

### Fecal metabolomics

A total of 140 metabolites were reliably detected in the current study (QC CV < 20%, 80% of sample signals > 1000). Cumulatively, 126 aqueous metabolites were reliably profiled using LC–MS/MS, while 14 short-chain fatty acids (SCFAs) were captured using GC–MS. A total of 13 missing values (0.2%) were detected and estimated using feature-wise *k*-nearest neighbors imputation. Data was normalized through square root transformation (square root of data values) and auto scaling (mean-centered and divided by the square root of the standard deviation of each variable); raw and normalized distribution are visualized in Supplementary Fig. S3.

Outlier detection was performed via random forest (RF) analysis using 500 decision trees. RF indicated five samples with a high magnitude of outlying measures (Supplementary Fig. S4). Of these, one sample was from the FI group (DW25), and four samples were from the FS group (DW10, DW100, DW52, DW96). These samples were removed from all subsequent metabolomic analyses.

**Figure 5:**
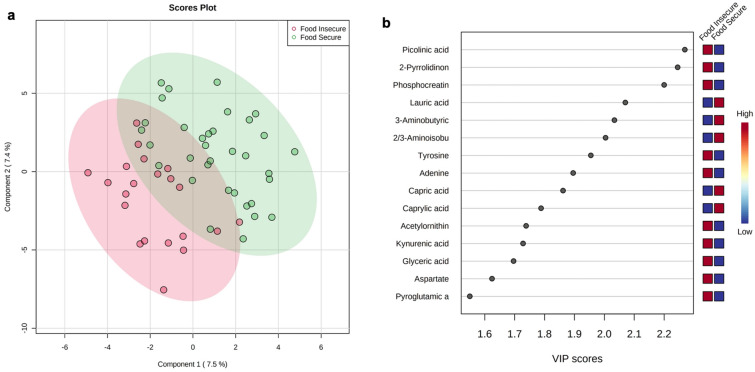
Partial least squares-discriminant analysis (PLS-DA) and variable importance in projection (VIP) analysis of food secure and food insecure groups using all detected metabolites in the study. (**a**) Two-dimensional scores plot of PLS-DA (*R*^2^*X* = 0.533, *R*^2^*Y* = 0.387, *Q*^2^ = − 0.340). (**b**) VIP scores of modeled metabolites between groups; top 15 metabolites are shown.

Principal component analysis (PCA) was performed using the entire set of reliably detected metabolites to assess global differences between groups. A two-dimensional scores plot is shown in Supplementary Fig. S5. The first two components account for approximately 25.2% of total variance, suggesting food security may exhibit low to moderate effects on the fecal metabolome. A supervised partial least squares-discriminant analysis (PLS-DA) multivariate model was estimated using all reliably detected metabolites (Fig. 5). The two-dimensional scores plot showed appreciable separation between FS and FI groups, although the first two components accounted for only ~ 15% of total variance (Fig. 5a). Variable importance in projection (VIP) scores were derived from the PLS-DA model and, while 50 metabolites had VIP > 1, six metabolites showed VIP > 2 (Fig. 5b): picolinic acid (VIP = 2.269), 2-pyrrolidinone (VIP = 2.245), phosphocreatine (VIP = 2.200), lauric acid (VIP = 2.070), 3-amino butyric acid (VIP = 2.034), and 2/3-aminoisobutyric acid (VIP = 2.004). Results of the PLS-DA and VIP analysis show that gross differences between groups are primarily accounted for by a relatively small subset of metabolites.

**Figure 6:**
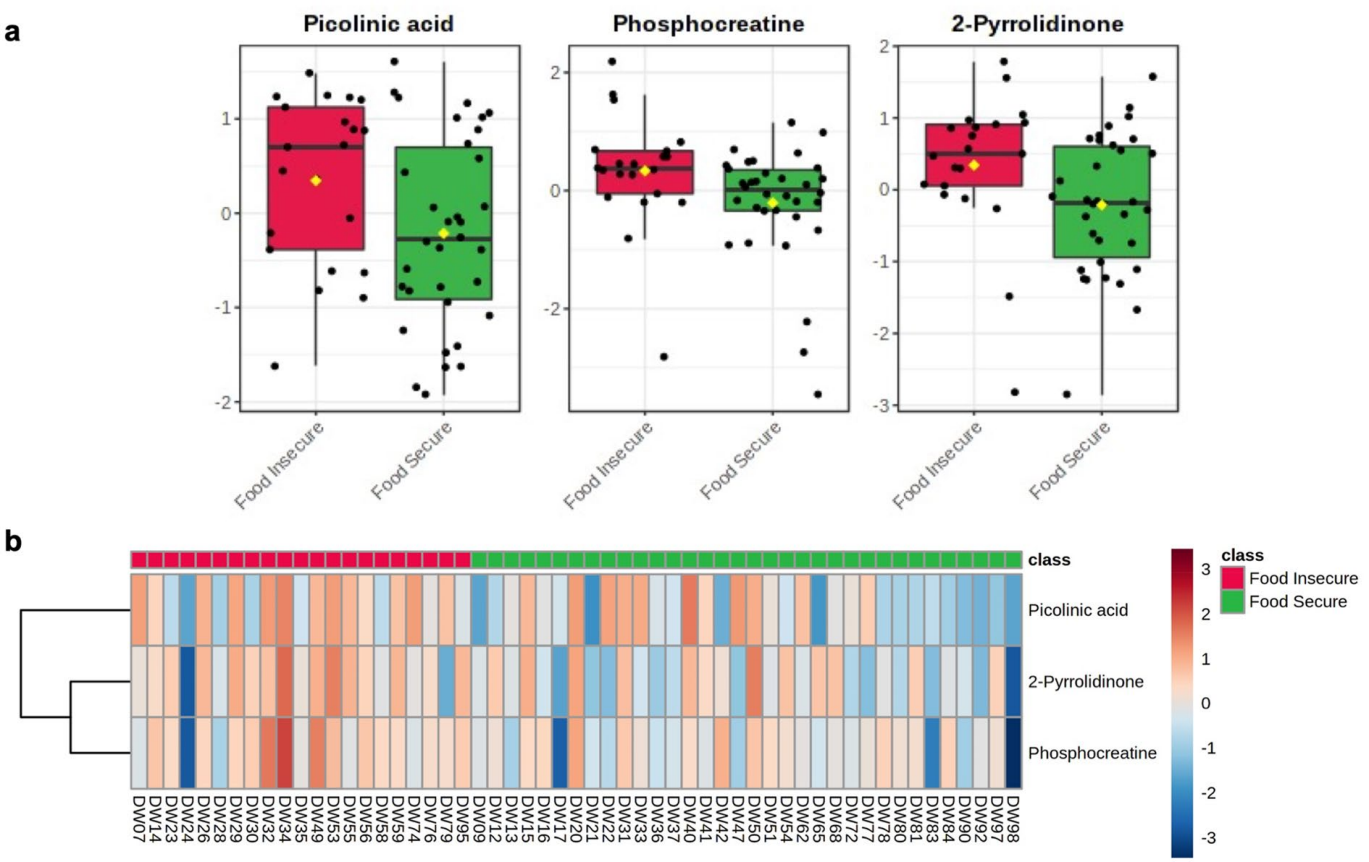
Results of general linear model (GLM) of significant fecal metabolites by food security status. Depression, sex, fiber intake, MVPA and BMI were controlled for as covariates. Equal variances were assumed and parametric testing was performed with FDR adjustment. Data are presented as normalized metabolite concentrations. (**a**) Box plots of significant between-group metabolites: picolinic acid (*q* = 0.043), phosphocreatine (*q* = 0.045), and 2-pyrrolidinone (*q* = 0.049). (**b**) Heatmap of significant metabolites for each participant between study groups.

To evaluate changes in individual metabolites between groups, an FDR-corrected general linear model (GLM) was constructed and depression, sex, fiber intake, MVPA and BMI were controlled for as covariates. While none of the monitored SCFAs were significantly altered between study groups, three aqueous metabolites were identified as significant between groups: picolinic acid (*q* = 0.043), phosphocreatine (*q* = 0.045), and 2-pyrrolidinone (*q* = 0.049). Box plots of significant metabolites are presented in Fig. 6a. A heatmap of significant metabolites between groups is visualized in Fig. 6b. Notably, these three metabolites also displayed the highest VIP scores as derived from PLS-DA and provided the highest relative contribution to model accuracy (Fig. 5).

Metabolomics data were also used to analyze predicted functional profiles. Disease enrichment analysis was performed using 44 metabolite sets reported in human feces, while enzyme enrichment analysis was performed using a library of 912 metabolite sets predicted to change in the case of dysfunctional enzymes; meanwhile, pathway analysis was performed by mapping detected metabolites to the KEGG human database. Significance of enrichment ratios was calculated via LASSO regression, whereas significance of pathway impact was calculated via a global test of relative-betweenness centrality. Results of disease and enzyme enrichment analyses are shown in Supplementary Fig. S6a and S6b, respectively, while the pathway analysis results are given in Supplementary Fig. S7. Although non-significant, a reduction in fecal metabolites related to enthesitis-related arthritis was observed in the FI group (*p* = 0.245) as well as a reduction in metabolites related to tyramine- and tyrosine-related sulfate exchange and carboxylase enzymes, among others (all *p* = 0.082). The ubiquinone and other terpenoid-quinone biosynthesis pathway was most significantly impacted (*p* = 0.082, impact = 0.0), while phenylalanine, tyrosine, and tryptophan biosynthesis showed the highest magnitude of pathway impact (*p* = 0.191, impact = 1.0).

### Integrative analysis of fecal microbes and metabolites differs by food security status

As an exploratory analysis, taxa at the genus level were integrated with metabolite features by FS status to assess potential co-occurrence patterns between the fecal microbiome and metabolome. In FI individuals, we found taxa with the greatest probability of co-occurrence were [*Clostridium*]* innocuum group*, *Lachnospiraceae FCS020 group*, *Lachnospiraceae UCG-008*, *Oscillospiraceae* (family level), and *Ruminococcaceae* (family level). These most co-occurred with the amino acids, L_alloisoleucine_leucine_norleucine (mmvec rank ≥ 4.164), isoleucine (mmvec rank ≥ 3.515), valine (mmvec Rank ≥ 3.348), and phenylalanine (mmvec rank ≥ 3.348; Supplementary Table S4). In comparison, taxa with the greatest probability of co-occurrence with fecal metabolites in FS individuals were *Chloroplast*, [*Clostridium*]* innocuum group*, *GCA-900066575*, *Lachnospiraceae FCS020 group*, and *Lachnospiraceae_UCG-008*. Despite differences in taxa, co-occurrence patterns displayed some similarities with the amino acids, Isoleucine (mmvec rank ≥ 4.104), L_alloisoleucine_leucine_norleucine (mmvec rank ≥ 3.711), and valine (mmvec rank ≥ 3.585). Unique co-occurrences in FS individuals included creatine, pyroglutamic_acid, and carnosine (mmvec rank ≥ 2.673).

## Discussion

This study sought to examine the association between food security status and the microbial and metabolomic signatures of the GM among a diverse set of emerging adults in college. We observed modest differences in beta and alpha diversity metrics by food security status that demonstrated FI students had greater microbial richness and evenness (within-sample differences). Further, FI students had a greater abundance of taxa *Enterobacteriaceae* and *Eisenbergiella*, whereas FS students had greater abundance of *Clostridia*, *Megasphaera*, and *Holdemanella*. FI students had greater predicted metabolic pathway activity for hydrolysis reactions, energy substrate biosynthesis, and macronutrient metabolism. Interestingly, three significant between-group metabolites were observed that related to energy turnover and the nervous system, including picolinic acid, phosphocreatine, and 2-pyrrolidinone. Taken together, these findings set the stage for a more expansive longitudinal approach, to examine the temporal relationship between food security status and the GM, and how this relationship may ultimately affect host health.

Tying host health to features of the GM has traditionally been conducted with high-level community metrics like alpha diversity. Broadly, this class of ecological measures has been considered a reflection of a health-associated gut microbiota. Indeed, previous research has reported that greater alpha diversity is indicative of better diet quality^33^ whereas food insecurity tends to result in lower dietary quality^2,22^. However, in the context of food security status it is not presently clear if the greater diversity reported in the FI students is a manifestation of a health-associated gut. For instance, greater levels of alpha diversity have been reported to be positively correlated with constipation and potentially toxic protein fermentation products^34,35^. Relatedly, we noted a predicted functional increase in adenosylcobinamide hydrolase (an enzyme which acts on peptide bonds) and co-occurrence patterns with fecal amino acids in FI participants. Despite differences in microbial diversity, the current study did not find differences in carbohydrate, sugar, fiber, protein, or fat intake by food security status. Unfortunately, we did not capture fluctuations in food consumption, though preclinical work has provided evidence that periods of food insecurity may promote reduced feeding and modify food preference behavior (i.e., seeking high fat foods)^36^. Fluctuating food availability has also been observed among hunter-gatherer populations due to seasonal variation (e.g., greater availability of high-fiber fruits and vegetables during the summer), which has resulted in season-dependent shifts in microbial community composition^37^. Thus, food insecurity may impact the GM in a similar manner.

In relation to microbial profiling, there is limited research that has explored the impact of food insecurity on the GM composition. A large birth cohort found that infants from FI households had significantly greater relative abundance of several genera from the *Lachnospiraceae* family including *Blautia* and *Dorea*, after adjustment for delivery method, breastfeeding status, and timing of introduction to solid foods^38^. Although the current study did not observe an increase in these genera, we did find an increased abundance of *Eisenbergiella* among FI students, another genera of the *Lachnospiraceae* family. Similarly, previous research has demonstrated that Ramadan-associated intermittent fasting has led to major shifts in microbiome composition including increased abundance of families *Enterobacteriaceae* and *Lachnospiraceae*^39^. The current study also found FI students to have an increased abundance of family *Enterobacteriaceae*. Bacteria belonging to family *Lachnospiraceae* ferment plant polysaccharides into SCFAs and alcohols but also are able to degrade mucin, which may be advantageous during periods of energy deprivation^40^, such as with food insecurity. Conversely, *Enterobacteriaceae* is a bacterial family that includes numerous pathogenic bacteria such as *Salmonella* and *Escherichia coli*, but also primarily consists of facultative anaerobes that ferment glucose^41^. Findings thus far suggest that altered food availability influences the ecological landscape of the GM, potentially increasing microbial competitiveness favoring taxa capable of metabolizing intestinal barrier components during periods of food insecurity.

Students classified as food secure in the present study had greater abundance of genera positively associated with carbohydrate metabolism. For instance, *Megasphaera* is a known producer of SCFAs^42^ and associated with glucose control in medicated patients with diabeties^43^. Species within the *Holdemanella* genus have been noted to promote anti-inflammatory activity in a mouse model for colitis^44^ and enhance GLP-1 signaling and improve glucose tolerance in obese mice^45^. However, as a genus, *Holdemanella* has been noted to have sex-specific associations between the gut microbiome and fat distribution with greater abundance being positively associated with the android fat ratio in males, and negatively associated in females^46^. While we did control for sex, it is important to highlight that nearly 70% of our participants were female. While difficult to parse out potential sex-specific microbial and metabolomic signatures in the current work, such efforts will be important considerations for future research.

Food insecurity has been associated with eating disorders such as anorexia and bulimia nervosa^28,47^. Unlike the findings from the current study, individuals with restrictive and binge-purge anorexia had unchanged^25^ or reduced bacterial richness^47^. Although unique microbial profiles have been reported among anorexics^25,47,48^, there were no similarities with the current study. Interestingly, one study found that after 3-days of underfeeding, individuals had a significant increase in total colonization levels with bacterial diversity increasing on the final day of underfeeding^24^. Given that the current study was unable to determine food security status at the exact time of fecal collection^24^, the increased GM diversity found might indicate short-term food insecurity similar to what was found during short-term periods of underfeeding.

Another potential explanation is that the primary driver of the observed GM differences in the current study may not be related to food security status but instead potential psychosocial issues pertinent to food insecurity. For example, previous research from our group demonstrated that food insecure college students had a three-fold higher odds of depression^9^. In turn, there is emerging evidence of a relationship between the gut microbiota and mental health^49^. Although the current study did not find differences in depression or stress by food security status, these psychosocial factors that are more prevalent among food insecure college students may partially explain the observed microbial differences. More research in this area is greatly warranted, particularly utilizing established clinical diagnostics in tandem with systems biology approaches such as blood and fecal metabolomics.

As incorporated in the present study, fecal metabolite analyses in GM investigations provide a critical window into metabolic function and offer insight into corresponding health implications for the host. Our analysis revealed FI students had elevations in picolinic acid, 2-pyrrolidinone, and phosphocreatine. Picolinic acid is an intermediate of the tryptophan-kynurenine pathway. Tryptophan is an important precursor for neurotransmitters serotonin and melatonin and the activity of this pathway may relate to the greater experience of depression among the FI group. The kynurenine pathway has been reviewed by experts in relation to its role in neurological health^50^. Picolinic acid has also been shown to regulate immune and inflammatory responses, both in the gut and systemically, through IFN-γ-dependent inducible nitric oxide synthase (iNOS) expression^51^. Food insecurity is also associated with increased inflammation, as evidenced by higher C-reactive protein concentrations in serum^52^. Further, 2-pyrrolidinone is a metabolite of the glutamine-glutamate/GABA pathway and recent work has suggested the importance of this pathway in gut–brain signaling via the GM^53^. A metagenomic analysis of 1054 individuals participating in the Flemish Gut Flora Project supports this theory with data showing that the gut microbiome alters the GABA pathway and is linked to both depression and decreased quality of life^54^. In the context of the aforementioned mood disturbance that occurs during periods of food insecurity, elevated levels of these molecules may indicate changes in gut–brain-axis and neurological cell communication. Such findings provide precedence for future longitudinal work to better characterize these potential changes in relation to the brain and other relevant organ systems.

Phosphocreatine was also an elevated fecal metabolite among FI students. This molecule is important to the central nervous system but less as a signaling molecule and more as an energy exchange molecule for its ability to convert ADP to ATP. Interestingly, an experimental study of food insecurity in European starling birds suggested that 1–2 weeks of limited and unpredictable food access resulted in more efficient energy extraction from foods consumed (body mass maintained per unit of food eaten)^55^. FI birds gained excess weight in a similar fashion to humans and the greater energy extraction persisted for 1–2 weeks after food insecurity was arrested. Alterations in phosphocreatine metabolism may also help to explain known links between food insecurity and depression^9^. Among germ-free mice that received intestinal microbiome transplants from humans with major depressive disorder phosphocreatine was significantly elevated in liver tissue compared to healthy control recipients^56^. This may be indicative of the role of mitochondria dysfunction in depression^57^. Taken together, phosphocreatine may be an important fuel source in times of food insecurity, helping to coordinate GM energy extraction and maintenance of critical neurological tissues.

Results from our integrative analysis of microbial features and metabolites revealed some similarities in co-occurrence patterns, including several amino acids and taxa from the *Lachnospiraceae* family. However, FI status displayed distinction by having a greater probability of co-occurrence of the select amino acids, including phenylalanine, with *Clostridium*, *Oscillospiraceae*, and *Ruminococcaceae*. Phenylalanine has been identified as a fecal metabolite with a connection to the brain^58^ and associated with GM disturbance in major depressive disorder^59^. In contrast, FS individuals had unique co-occurrences with creatine and carnosine, and the glutamic acid derivative, pyroglutamic acid. Of note, pyroglutamic acid has been reported to be decreased in the fecal metabolome of Parkinson’s disease^60^. Reduction in glutamic acid has been suggested to mirror oxidative stress associated with neurological degeneration^61^. These results further reinforce the potential gut–brain signaling via the GM, though more research is needed to better understand the implications of these differences. Finally, per the multiple constraints in the current analysis, great caution is warranted in interpretation of these results and GM correlation-type analyses in general^62^.

This study engaged a diverse sample of college students at a large public institution. Study findings are novel and provide context for future research but may not be generalizable to a non-college student sample. This is partly evident in the relatedness of weight status of participants in the present analysis. While the literature is relatively inconsistent when examining the relationship between food insecurity and BMI^63–66^, we acknowledge that the sample of participants in the current study may not be representative of food insecurity over the spectrum of weight status. In addition, as our study participants were students, fully capturing economic condition was a limitation. We used Pell grant status as a proxy and, while perhaps an imperfect metric, we noted no significant difference between classifications. The food security measurement has been validated among low-income adults, but not emerging adults^6^. Although the current study collected stool samples either the same day, or no more than 5 days post food security questionnaire completion, the questionnaire used assessed food insecurity within the last 30 days. There are no validated questionnaires that assess more recent food security status which may be problematic when drawing inferences about GM composition. However, the effects of food insecurity may persist for a few weeks after the re-establishment of food security, as evidenced in animal models^55^, making it more likely that we captured relevant metabolomic and GM indicators of food insecurity. As a cross-sectional study**,** we were unable to examine the causal relationship between food insecurity and the GM. In relation, results from our integrative analysis of fecal microbial features and metabolites suffered from multiple constraints, including taxonomic resolution and functional description obtained from 16S sequencing. Therefore, caution is warranted in interpretation of these results based on our limited number of participants and the correlational nature of our GM-metabolome analysis. Moreover, we did not restrict participant inclusion based on alcohol consumption, which may have influenced our findings. However, we did not detect a significant difference in weekly alcohol consumption by food security classification. Lastly, the assessments of diet and physical activity were based on self-report, which are prone to recall and social desirability bias.

In conclusion, although the current study found some unique differences in bacterial community structure and metabolite production by food insecurity status, the differences were subtle. Our findings provide evidence to support the resiliency of gut microbiota during periods of limited caloric intake or reduced dietary diversity but also suggest potential links between GM disruption, altered metabolism and food insecurity. Future studies should corroborate these findings with larger sample sizes and apply them to longitudinal assessments of food security status with more robust microbial and metabolite sampling.

## Methods

### Study design

This is a secondary analysis of a cross-sectional pilot study that examined the impact of social networks on college student nutrition, physical activity, and weight outcomes^67^. Participants were recruited from three residence halls across one urban campus. Once eligible students were enrolled in the primary study (*n* = 221), they were given the opportunity to enroll in the devilWASTE study (*n* = 60). The exclusion criteria for devilWASTE included being under the age of 18, certain gastrointestinal conditions such as malabsorptive disease, history of an eating disorder, antibiotic use 2–3 months prior, and current conditions (diagnosed and/or treated) that affect the microbiome including HIV infection, diabetes, or high blood pressure. Any medications reported by participants were evaluated individually for their ability to robustly influence the gut microbiome, in which case a participant was excluded from participating in the devilWASTE study. Inclusion criteria were living in a residence hall at ASU, English speaking, and participation in SPARC study. Participants provided informed written consent and all study protocols were approved by the Arizona State University Institutional Review Board (STUDY00005882). Additionally, all methods were performed in accordance with the relevant guidelines and regulations.

### Food insecurity

Food insecurity was measured using an adaptation of the 2-item food insecurity screener^68^. The time frame in the validated question was adapted and changed the framing of the question from “we” to “I”, as has been done by others^69^. Participants were asked, “Within the past month, I worried whether my food would run out before I got money to buy more” and “Within the past month, the food I bought just did not last and I did not have money to get more.” Students giving an affirmative answer to either question were categorized as food insecure in the past month.

### Fecal sample collection and DNA extraction

Research staff delivered fecal sample collections kits to the residence halls of eligible participants. Fecal samples were collected at a single timepoint and participants were asked to report any medication and supplement use within the last 3 months. If participants had taken any antibiotics, antifungals, or probiotics within the previous 3 months, a fecal sample was not obtained. Research staff picked up the fecal samples within 30 min of participate reported bowel movement and transported them to the laboratory where they were frozen at − 80 °C until further processing. Frozen samples were thawed at 4 °C, and wet weight was recorded to the nearest 0.01 g after subtracting the weight of fecal collection materials. DNA was extracted from approximately 300 mg of feces, collected from the center of the sample, using a modified version of the manufacturer protocol (MoBio Power Soil DNA Isolation Kit #12888-100, MoBio, Carlsbad, CA). Per manufacturer recommendations, a heating step of 65 °C for 10 min was added to the protocol to reduce the influence of inhibitors commonly found in feces and increase DNA yield. DNA concentration and quality were quantified using QIAxpert System (Qiagen, Germantown, MD) according to manufacturer instructions.

### Analyses

#### Fecal microbiome sequencing and statistical analysis

High-throughput genomic sequencing of the 16S rRNA gene was performed using Illumina miSeq technology after ligating 515F and 806R primers and Illumina adapters via polymerase chain reaction. Negative controls were included and run with the study samples. A detailed report of methods to prepare and sequence DNA has been published^18^. Raw 16S rRNA sequencing data for all samples have been deposited in the open-source repository “NCBI/Sequence Read Archive (SRA)” under project PRJNA473006 with accession numbers: SAMN09258197–SAMN09258278 (https://www.ncbi.nlm.nih.gov/sra).

Overall, the 16S rRNA sequencing produced 5,259,656 reads with a median of 80,443 reads per sample (per-sample sequence count range: 20,558–197,883). Paired-end, demultiplexed data were imported and analyzed using QIIME 2 software version 2021.8. Upon examination of sequence quality plots, base pairs were trimmed at position 13 and truncated at position 150 and were run through DADA2 to remove low quality regions and construct a feature table using ASVs (Supplemental Fig. S8). Next, the ASV feature table was passed through the feature-classifier plugin, which was implemented using a naive Bayes machine-learning classifier, pre-trained to discern taxonomy mapped to the latest version of the rRNA database SILVA (138.1; 99% OTUs from 515F/806R region of sequences)^70^. Based on assessment of alpha rarefaction (p-min-depth = 10 and p-max-depth = 120,000) a threshold of 22,000 sequences/sample was established leaving 58/60 high quality samples (participants DW09 and DW96 were removed). A phylogenic tree was then constructed using the fragment-insertion plugin at a p-sampling depth of the rarefaction threshold to impute high-quality reads and normalize for uneven sequencing depth between samples.

Diversity analyses were conducted with the diversity plugin. Alpha diversity (intra-community diversity) was measured using richness (Shannon, Faith’s PD and observed features) and evenness (Pielou’s E) indexes. Beta diversity (inter-community diversity) was measured using Jaccard, Bray–Curtis, Unweighted UniFrac distance (qualitative measure), and Weighted UniFrac distance (quantitative measure). GLM and Adonis analyses were used to test for significant differences (alpha = 0.05) between FS and FI status for alpha and beta diversity metrics, respectively. Both statistical models incorporated the covariates sex, BMI, fiber intake, and self-reported depression and MVPA.

Differential abundance was calculated using Songbird (v1.0.126) in QIIME 2^29^. Specifically, differentials were computed (parameters: –p-epochs 10,000 –p-differential-prior 0.5 –p-summary-interval 1 –num-random-test-examples 10% of samples) based on FS and FI status and accounting for the covariates sex, BMI, fiber intake, and self-reported depression and MVPA. Qurro (v0.4.027) was then used to compute log ratios of ranked features^30^. Evaluation of the Songbird models against a baseline model obtained a pseudo-*Q*^2^ value of 0.183. The top 10 lowest and highest ranked differential features were selected and a Mann–Whitney *U* test and Cohen’s d were calculated to assess the significance (alpha = 0.05) and effect size of the log ratios.

To examine the correlation between taxa at the genus level by food security status the SparrCC algorithm using FastSpar was implemented to render a network analysis with a correlation threshold of 0.6 or − 0.6 (# of permutations = 99; alpha = 0.05)^31^. Importantly, SparCC assumes network sparsity and uses a log-ratio transformation, performing iterations to identify taxa pairs that are outliers to background correlations. Next, correlation pattern searches were used for the dominant taxa, with individual correlations assessed using a FDR correction (*q* < 0.05). Both a correlation network analysis and correlation pattern search were employed using MicrobiomeAnalyst^71^.

The PICRUSt 2 pipeline^32^ was implemented in order to predict the function of fecal microbiota. Output for the level 3 of the KEGG were analyzed using Songbird (v1.0.126) in QIIME 2 as previously described for the differential abundance analysis. Evaluation of the Songbird models against a baseline model obtained a pseudo-*Q*^2^ value of 0.210. The top 10 lowest and highest ranked differential features were selected and a Mann–Whitney *U* test and Cohen’s *d* were calculated to assess the significance (alpha = 0.05) and effect size of the log ratios.

#### Metabolomics analysis

LC–MS grade acetonitrile (ACN), methanol (MeOH), and ammonium acetate (NH_4_Oac) were purchased from Fisher Scientific (Pittsburgh, PA), while ammonium hydroxide (NH_4_OH), *O*-methylhydroxylamine hydrochloride (MeOX), and *N*-Methyl-*N*-(tert-butyldimethylsilyl) trifluoroacetamide (MTBSTFA) were bought from Sigma-Aldrich (Saint Louis, MO). Deionized water was sourced in-house by a water purification system from EMD Millipore (Billerica, MA). Phosphate buffered saline (PBS) was procured from GE Healthcare Life Sciences (Logan, UT). Standard compounds corresponding to measured aqueous metabolites were purchased from Sigma-Aldrich and Fisher Scientific. Lipid standards used in this study were purchased from Fisher Scientific, Sigma-Aldrich, and Avanti Polar Lipids (Alabaster, AL).

Prior to LC–MS/MS targeted analysis, frozen fecal samples were first thawed overnight under 4 °C. Afterward, 20 mg of each sample were placed in a 1.5 mL Eppendorf vial. Protein precipitation and metabolite extraction was performed by adding 500 μL MeOH and 50 μL internal standard solution (containing 1810.5 μM ^13^C_3_-lactate and 142 μM ^13^C_5_-glutamic acid). The mixture was then vortexed for 10 s and stored at − 20 °C for 30 min; afterward, samples were centrifuged at 14,000 RPM for 10 min at 4 °C. The supernatants (450 μL) from these samples were collected into new Eppendorf vials and dried using a CentriVap Concentrator (Fort Scott, KS). Dried samples were then reconstituted in 150 μL of 40% PBS/60% ACN and centrifuged again at 14,000 RPM at 4 °C for 10 min. Finally, 100 μL of supernatant was collected from each sample into an LC autosampler vial for subsequent analysis. A pooled sample, which was a mixture of all experimental samples, was used as the quality control (QC) sample and injected once every 10 experimental samples.

The targeted LC–MS/MS method used here is detailed elsewhere^72,73^. Briefly, all LC–MS/MS experiments were performed using an Agilent 1290 UPLC-6490 QQQ-MS system. Each sample was injected twice, 10 µL for analysis using negative ionization mode and 4 µL for analysis using positive ionization mode. Both chromatographic separations were performed in hydrophilic interaction chromatography (HILIC) mode on a Waters Xbridge BEH Amide column (150 × 2.1 mm, 2.5 µm particle size; Waters Corporation, Milford, MA). The HILIC parameters were as follows: flow rate was 0.3 mL/min, auto-sampler temperature was kept at 4 °C, and the column compartment was set to 40 °C. The mobile phase for LC separations was composed of Solvents A (10 mM NH_4_Oac, 10 mM NH_4_OH in 95% H_2_O/5% ACN) and B (10 mM NH_4_Oac, 10 mM NH4OH in 95% ACN/5% H_2_O). After an initial 1 min isocratic elution of 90% B, the percentage of Solvent B decreased to 40% at t = 11 min and was maintained at 40% for 4 min (t = 15 min), after which the percentage of B gradually went back to 90%, to prepare for the next injection. The mass spectrometer was equipped with an electrospray ionization (ESI) source and targeted data acquisition was performed in multiple-reaction-monitoring (MRM) mode. All aspects of the LC–MS system was controlled by Agilent MassHunter Workstation software. Subsequently, the extracted MRM peaks were integrated using Agilent MassHunter Quantitative Data Analysis software.

Prior to GC–MS analysis of SCFAs, frozen fecal samples were first thawed overnight under 4 °C. Then, 20 mg of each sample was homogenized with 5 μL hexanoic acid-3,3,3 (internal standard), 15 μL sodium hydroxide (NaOH [0.5 M]), and 500 μL MeOH. Samples were then stored at − 20 °C for 20 min and centrifuged afterward at 14,000 RPM for 10 min. Next, 450 μL of supernatant were collected and sample pH was adjusted to 10 by adding 30 μL of NaOH:H_2_O (1:4, v:v). Samples were then dried, and the residues were initially derivatized with 40 µL of 20 mg/mL MeOX solution in pyridine under 60 °C for 90 min. Subsequently, 60 µL of MTBSTFA containing d_27_-mysristic acid were added, and the mixture was incubated at 60 °C for 30 min. The samples were then vortexed for 30 s and centrifuged at 14,000 RPM for 10 min. Finally, 70 µL of supernatant were collected from each sample and injected into new glass vials for GC–MS analysis.

GC–MS conditions used here were adopted from a previously published protocol^74,75^. Briefly, GC–MS experiments were performed on an Agilent 7820A GC-5977B MSD system (Santa Clara, CA); all samples were analyzed by injecting 1 µL of prepared samples. Helium was the carrier gas with a constant flow rate of 1.2 mL/min. Separation of metabolites was achieved using an Agilent HP-5 ms capillary column (30 m × 250 µm × 0.25 µm). Ramping parameters were as follows: column temperature was maintained at 60 °C for 1 min, increased at a rate of 10 °C/min to 325 °C, and then held at this temperature for 10 min. Mass spectral signals were recorded at an *m/z* range of 50–600 and data extraction was performed using Agilent Quantitative Analysis software.

Following peak integration, metabolites were filtered for reliability and only those with QC CV < 20% and relative abundance of 1000 in > 80% of samples were retained for statistical analysis. The acquired data were then square root transformed and auto scaled prior to analysis. Linear modelling was performed using SPSS 28.0 (SPSS Inc., Chicago, IL), while multivariate statistical analyses were performed using open-source R software.

#### Microbiome and metabolome integration and co-occurrence analysis

Probabilities of cooccurrence between fecal taxa and metabolites was conducted using mmvec (v1.0.2), a neural network solution inspired from natural language processing^76^. A log-transformed conditional probability matrix from each cross-omics feature pair was constructed and a singular value decomposition was applied in order to represent cooccurrence. These are displayed as rank values in Supplementary Table S4.

### Covariates

There were several covariates that were accounted for in our analyses based on previous studies demonstrating a significant impact on gut microbiota composition.

#### Demographic data

Participant gender was provided via a self-reported, web-based questionnaire that was completed upon entry to the parent study. Participants were asked their sex. Height and weight measurements were obtained using Seca stadiometers (model 217) and Seca flat scales (model 874 or 869, respectively, by trained research staff. Measures were taken in triplicate with the two closest values (i.e., within 0.5 cm and 0.5 kg) averaged. The averaged values were used to calculate BMI to the nearest kg/m^2^. CDC BMI guidelines were used to categorize participants as normal weight (BMI ≥ 18.5 kg/m^2^ and ≤ 24.9 kg/m^2^), overweight (BMI ≥ 25.0 kg/m^2^ and ≤ 29.9 kg/m^2^), or obese (BMI ≥ 30.0 kg/m^2^).

#### Depression

Students were asked “How often in the past 1 month have you felt: (1) Things were hopeless?; (2) Overwhelmed by all you had to do?; (3) Very lonely?; (4) Very sad?; (5) So depressed that it was difficult to function?; (6) Overwhelming anxiety? (Response options: never, rarely, sometimes, often)”.

#### Fiber intake

The ASA24 24-h dietary recall was used to assess students’ habitual dietary intake. Food and beverage intake was recorded from midnight to midnight on the previous day. Participants were asked to complete 3 days of dietary recall (2 weekdays and 1 weekend day) which has been previously validated^77,78^. The ASA24 uses the U.S. Department of Agriculture’s Automated Multiple Pass Method (AMPM)^79^ and measures intake by using the USDA’s Food and Nutrient Database for Dietary Studies (FNDDS). Using data from the ASA24-2014, we examined total fiber intake.

#### Physical activity

Physical activity habits were determined using the Godin-Shephard Leisure-Time Physical Activity Questionnaire which has been validated as an appropriate method to measure physical activity habits among college students^80^. The amount of time participants’ spent engaged in moderate and vigorous exercise was obtained by asking “In a usual week, how many hours do you spend doing the following activities:” with the question endings of strenuous, moderate, and mild exercise. Response options ranged from “None” to “more than 6 h per week”. The amount of moderate-to-vigorous physical activity was calculated by totaling the time reported in moderate and strenuous exercise.

### Ethics statement

This study included the participation of human volunteers. All protocols and consenting procedures were reviewed and approved by the Arizona State University Institutional Review Board. Written informed consent was obtained from all participants and a copy of the signed consent form was provided to each participant for their records. Each participant was informed that their participation was voluntary and could be stopped at any time. Identifying information (e.g., names) for participants have been removed from all text, figures, tables, and images to ensure their anonymity.

## Supplementary Information

Supplementary Information.

## Data Availability

Raw 16S rRNA sequencing data for all samples have been deposited in the open-source repository “NCBI/Sequence Read Archive (SRA)” under project PRJNA473006 with accession numbers: SAMN09258197–SAMN09258278 (https://www.ncbi.nlm.nih.gov/sra). All mass spectrometry data and deidentified subject metadata analyzed in this study have been deposited to Mendeley Data and are publicly available (10.17632/s4ydf6kb8y.1).

## References

[1] Coleman-Jensen, A., Rabbitt, M. P., Gregory, C. & Singh, A. Household food security in the United States in 2014. *US Household Food Security: Statistics and Analysis for 2014*, 1–56. 10.2139/ssrn.2504067 (2016).

[2] Hanson KL, Connor LM (2014). Food insecurity and dietary quality in US adults and children: A systematic review. Am. J. Clin. Nutr..

[3] Gundersen C, Seligman HK (2015). Food insecurity and health outcomes. Health Aff..

[4] Kwan MY, Cairney J, Faulkner GE, Pullenayegum EE (2012). Physical activity and other health-risk behaviors during the transition into early adulthood: A longitudinal cohort study. Am. J. Prev. Med..

[5] Bruening M, Argo K, Payne-Sturges D, Laska MN (2017). The struggle is real: A systematic review of food insecurity on postsecondary education campuses. J. Acad. Nutr. Diet..

[6] Ellison B, Bruening M, Hruschka DJ (2021). Viewpoint: Food insecurity among college students: A case for consistent and comparable measurement. Food Policy.

[7] Larson N, Laska MN, Neumark-Sztainer D (2020). Food insecurity, diet quality, home food availability, and health risk behaviors among emerging adults: Findings from the EAT 2010–2018 study. Am. J. Public Health.

[8] Van Woerden I, Hruschka D, Vega-López S, Schaefer DR, Adams M, Bruening M (2019). Food insecure college students and objective measurements of their unused meal plans. Nutrients.

[9] Bruening M, Brennhofer S, van Woerden I, Todd M, Laska M (2016). Factors related to the high rates of food insecurity among diverse, urban college freshmen. J. Acad. Nutr. Diet..

[10] Phillips E, McDaniel A, Croft A (2018). Food insecurity and academic disruption among college students. J. Stud. Aff. Res. Pract..

[11] van Woerden I, Hruschka D, Bruening M (2019). Food insecurity negatively impacts academic performance. J. Public Aff..

[12] Robertson RC, Manges AR, Finlay BB, Prendergast AJ (2019). The human microbiome and child growth—First 1000 days and beyond. Trends Microbiol..

[13] Fan Y, Pedersen O (2021). Gut microbiota in human metabolic health and disease. Nat. Rev. Microbiol..

[14] Pimentel M, Lembo A (2020). Microbiome and its role in irritable bowel syndrome. Dig. Dis. Sci..

[15] Rieder R, Wisniewski PJ, Alderman BL, Campbell SC (2017). Microbes and mental health: A review. Brain Behav. Immun..

[16] Tremaroli V, Bäckhed F (2012). Functional interactions between the gut microbiota and host metabolism. Nature.

[17] Zheng X, Xie G, Zhao A (2011). The footprints of gut microbial-mammalian co-metabolism. J. Proteome Res..

[18] Whisner CM, Maldonado J, Dente B, Krajmalnik-Brown R, Bruening M (2018). Diet, physical activity and screen time but not body mass index are associated with the gut microbiome of a diverse cohort of college students living in university housing: A cross-sectional study. BMC Microbiol..

[19] Ortega-Santos CP, Bruening M, Whisner CM, Journey EK (2019). Changes in weight status and the intestinal microbiota among college freshman, aged 18 y. J. Adolesc. Health..

[20] Feng T, Hilal MG, Wang Y (2021). Differences in gut microbiome composition and antibiotic resistance gene distribution between Chinese and Pakistani university students from a common peer group. Microorganisms..

[21] Littlejohn P, Finlay BB (2021). When a pandemic and an epidemic collide: COVID-19, gut microbiota, and the double burden of malnutrition. BMC Med..

[22] Christian VJ, Miller KR, Martindale RG (2020). Food insecurity, malnutrition, and the microbiome. Curr. Nutr. Rep..

[23] Wang L, Solá DDÁ, Flores MA (2020). Prenatal food insecurity post Hurricane Maria is associated with decreased Veillonella in the infant gut. Pediatr. Res..

[24] Basolo A, Hohenadel M, Ang QY (2020). Effects of underfeeding and oral vancomycin on gut microbiome and nutrient absorption in humans. Nat. Med..

[25] di Lodovico L, Mondot S, Doré J, Mack I, Hanachi M, Gorwood P (2020). Anorexia nervosa and gut microbiota: A systematic review and quantitative synthesis of pooled microbiological data. Prog. Neuropsychopharmacol. Biol. Psychiatry.

[26] Dao MC, Everard A, Aron-Wisnewsky J (2016). *Akkermansia muciniphila* and improved metabolic health during a dietary intervention in obesity: Relationship with gut microbiome richness and ecology. Gut.

[27] Mohr AE, Gumpricht E, Sears DD, Sweazea KL (2021). Recent advances and health implications of dietary fasting regimens on the gut microbiome. Am. J. Physiol. Gastrointest. Liver Physiol..

[28] Hazzard VM, Loth KA, Hooper L, Becker CB (2020). Food insecurity and eating disorders: A review of emerging evidence. Curr. Psychiatry Rep..

[29] Morton JT, Marotz C, Washburne A (2019). Establishing microbial composition measurement standards with reference frames. Nat. Commun..

[30] Fedarko MW, Martino C, Morton JT (2020). Visualizing ’omic feature rankings and log-ratios using Qurro. NAR Genomics Bioinform..

[31] Watts SC, Ritchie SC, Inouye M, Holt KE (2019). FastSpar: Rapid and scalable correlation estimation for compositional data. Bioinformatics.

[32] Douglas G, Maffei V, Zaneveld J (2020). PICRUSt2 for prediction of metagenome functions. Nat. Biotechnol..

[33] Lozupone CA, Stombaugh JI, Gordon JI, Jansson JK, Knight R (2012). Diversity, stability and resilience of the human gut microbiota. Nature.

[34] Zhu L, Liu W, Alkhouri R (2014). Structural changes in the gut microbiome of constipated patients. Physiol. Genomics.

[35] Wilmanski T, Rappaport N, Earls JC (2019). Blood metabolome predicts gut microbiome α-diversity in humans. Nat. Biotechnol..

[36] Estacio SM, Thursby MM, Simms NC (2021). Food insecurity in older female mice affects food consumption, coping behaviors, and memory. PLoS One.

[37] Ecklu-Mensah G, Gilbert J, Devkota S (2022). Dietary selection pressures and their impact on the gut microbiome. Cell. Mol. Gastroenterol. Hepatol..

[38] Benjamin-Neelon S, Differding M, Mueller N (2019). Infants from food insecure households have altered gut microbiota (OR01-03-19). Curr. Dev. Nutr..

[39] Su J, Wang Y, Zhang X (2021). Remodeling of the gut microbiome during Ramadan-associated intermittent fasting. Am. J. Clin. Nutr..

[40] Larrick JW, Mendelsohn AR, Larrick JW (2021). Beneficial gut microbiome remodeled during intermittent fasting in humans. Rejuvenation Res..

[41] Rock C, Donnenberg MS (2014). Human pathogenic Enterobacteriaceae. Ref. Module Biomed. Sci..

[42] Shetty SA, Marathe NP, Lanjekar V, Ranade D, Shouche YS (2013). Comparative genome analysis of *Megasphaera* sp. reveals niche specialization and its potential role in the human gut. PLoS One.

[43] de La Cuesta-Zuluaga J, Mueller NT, Corrales-Agudelo V (2017). Metformin is associated with higher relative abundance of mucin-degrading *Akkermansia muciniphila* and several short-chain fatty acid-producing microbiota in the gut. Diabetes Care.

[44] Pujo J, Petitfils C, le Faouder P (2021). Bacteria-derived long chain fatty acid exhibits anti-inflammatory properties in colitis. Gut.

[45] Romaní-Pérez M, López-Almela I, Bullich-Vilarrubias C (2021). *Holdemanella biformis* improves glucose tolerance and regulates GLP-1 signaling in obese mice. FASEB J..

[46] Min Y, Ma X, Sankaran K (2019). Sex-specific association between gut microbiome and fat distribution. Nat. Commun..

[47] Monteleone AM, Troisi J, Serena G (2021). The gut microbiome and metabolomics profiles of restricting and binge-purging type anorexia nervosa. Nutrients.

[48] Mack I, Penders J, Cook J, Dugmore J, Mazurak N, Enck P (2018). Is the impact of starvation on the gut microbiota specific or unspecific to anorexia nervosa? A narrative review based on a systematic literature search. Curr. Neuropharmacol..

[49] Peirce JM, Alviña K (2019). The role of inflammation and the gut microbiome in depression and anxiety. J. Neurosci. Res..

[50] Davis I, Liu A (2015). What is the tryptophan kynurenine pathway and why is it important to neurotherapy?. Expert Rev. Neurother..

[51] Melillo G, Cox GW, Biragyn A, Sheffler LA, Varesio L (1994). Regulation of nitric-oxide synthase mRNA expression by interferon-gamma and picolinic acid. J. Biol. Chem..

[52] Gowda C, Hadley C, Aiello AE (2012). The association between food insecurity and inflammation in the US adult population. Am. J. Public Health.

[53] Baj A, Moro E, Bistoletti M, Orlandi V, Crema F, Giaroni C (2019). Glutamatergic signaling along the microbiota–gut–brain axis. Int. J. Mol. Sci..

[54] Valles-Colomer M, Falony G, Darzi Y (2019). The neuroactive potential of the human gut microbiota in quality of life and depression. Nat. Microbiol..

[55] Bateson M, Andrews C, Dunn J (2021). Food insecurity increases energetic efficiency, not food consumption: An exploratory study in European starlings. PeerJ.

[56] Li B, Guo K, Zeng L (2018). Metabolite identification in fecal microbiota transplantation mouse livers and combined proteomics with chronic unpredictive mild stress mouse livers. Transl. Psychiatry.

[57] Allen J, Romay-Tallon R, Brymer KJ, Caruncho HJ, Kalynchuk LE (2018). Mitochondria and mood: Mitochondrial dysfunction as a key player in the manifestation of depression. Front. Neurosci..

[58] Zhang S, Qian Y, Li Q (2021). Metabolic and neural mechanisms underlying the associations between gut bacteroides and cognition: A large-scale functional network connectivity study. Front. Neurosci..

[59] Yang J, Zheng P, Li Y (2020). Landscapes of bacterial and metabolic signatures and their interaction in major depressive disorders. Sci. Adv..

[60] Vascellari S, Palmas V, Melis M (2020). Gut microbiota and metabolome alterations associated with Parkinson’s disease. mSystems.

[61] Lei S, Zavala-Flores L, Garcia-Garcia A (2014). Alterations in energy/redox metabolism induced by mitochondrial and environmental toxins: A specific role for glucose-6-phosphate-dehydrogenase and the pentose phosphate pathway in paraquat toxicity. ACS Chem. Biol..

[62] Carr A, Diener C, Baliga NS, Gibbons SM (2019). Use and abuse of correlation analyses in microbial ecology. ISME J..

[63] Pourmotabbed A, Moosavian S, Hadi A (2020). The relationship between food insecurity and risk of overweight or obesity in under 18 years individuals: A systematic review and meta-analysis. Int. J. Prev. Med..

[64] Bruening M, van Woerden I, Todd M, Laska MN (2018). Hungry to learn: The prevalence and effects of food insecurity on health behaviors and outcomes over time among a diverse sample of university freshmen. Int. J. Behav. Nutr. Phys. Act..

[65] Knol LL, Robb CA, McKinley EM, Wood M (2017). Food insecurity, self-rated health, and obesity among college students. Am. J. Health Educ..

[66] el Zein A, Shelnutt KP, Colby S (2019). Prevalence and correlates of food insecurity among U.S. college students: A multi-institutional study. BMC Public Health.

[67] Bruening M, Ohri-Vachaspati P, Brewis A (2016). Longitudinal social networks impacts on weight and weight-related behaviors assessed using mobile-based ecological momentary assessments: Study protocols for the SPARC study. BMC Public Health.

[68] United States Department of Agriculture. U.S. Household Food Security Survey Module: Three-Stage Design, With Screeners. https://www.ers.usda.gov/topics/food-nutrition-assistance/food-security-in-the-us/survey-tools/#household. Accessed November 19, 2021

[69] Nikolaus CJ, Ellison B, Nickols-Richardson SM (2019). Are estimates of food insecurity among college students accurate? Comparison of assessment protocols. PLoS One.

[70] Quast C, Pruesse E, Yilmaz P (2013). The SILVA ribosomal RNA gene database project: Improved data processing and web-based tools. Nucleic Acids Res..

[71] Chong J, Liu P, Zhou G, Xia J (2020). Using MicrobiomeAnalyst for comprehensive statistical, functional, and meta-analysis of microbiome data. Nat. Protoc..

[72] Jasbi P, Shi X, Chu P (2021). Metabolic profiling of neocortical tissue discriminates Alzheimer’s disease from mild cognitive impairment, high pathology controls, and normal controls. J. Proteome Res..

[73] Basile AJ, Mohr AE, Jasbi P, Gu H, Deviche P, Sweazea KL (2020). A four-week high fat diet does not alter plasma glucose or metabolic physiology in wild-caught mourning doves (*Zenaida macroura*). Comp. Biochem. Physiol. Part A Mol. Integr. Physiol..

[74] Jasbi P, Baker O, Shi X (2019). Daily red wine vinegar ingestion for eight weeks improves glucose homeostasis and affects the metabolome but does not reduce adiposity in adults. Food Funct..

[75] Gu H, Jasbi P, Patterson J, Jin Y (2021). Enhanced detection of short-chain fatty acids using gas chromatography mass spectrometry. Curr. Protoc..

[76] Morton JT, Aksenov AA, Nothias LF (2019). Learning representations of microbe–metabolite interactions. Nat. Methods.

[77] Ma Y, Olendzki BC, Pagoto SL (2009). Number of 24-hour diet recalls needed to estimate energy intake. Ann. Epidemiol..

[78] Johnson RK, Driscoll P, Goran M (1996). Comparison of multiple-pass 24-hour recall estimates of energy intake with total energy expenditure determined by the doubly labeled water method in young children. J. Am. Diet. Assoc..

[79] Blanton CA, Moshfegh AJ, Baer DJ, Kretsch MJ (2006). The USDA automated multiple-pass method accurately estimates group total energy and nutrient intake. J. Nutr..

[80] Godin G, Shephard R (1985). A simple method to assess exercise behavior in the community. Can. J. Appl. Sci..

