# Supplementary material for: Association of food insecurity on gut microbiome and metabolome profiles in a diverse college-based sample
Source: Sci Rep. 2022 Aug 23;12:14358. doi: 10.1038/s41598-022-18515-y (PMC9399224; doi:10.1038/s41598-022-18515-y)
Supplement: Supplementary file 1 — Supplementary Information. [file 41598_2022_18515_MOESM1_ESM.pdf]

Supplementary Information

Association of food insecurity on gut microbiome and metabolome profiles in a diverse college-based sample

Alex E. Mohr, MS[1+]; Paniz Jasbi, PhD[1,2+]; Kiley B. Vander Wyst, PhD, MPH, RD[3]; Irene van Woerden, PhD, MS[4]; Xiaojian Shi, PhD[1,5], Haiwei Gu, PhD[1,6]; Corrie M. Whisner, PhD[1,7]*; Meg Bruening, PhD, MPH, RD[1]*

[1]College of Health Solutions, Arizona State University, Phoenix, AZ, USA

[2]School of Molecular Sciences, Arizona State University, Tempe, AZ, USA

[3]College of Graduate Studies, Midwestern University, Glendale, AZ, USA

[4]Community and Public Health, Idaho State University, Pocatello, ID, USA

[5]Systems Biology Institute, Yale University, West Haven, CT, USA

[6]Center for Translational Science, Florida International University, Port St. Lucie, FL, USA

[7]Biodesign Institute Health Through Microbiomes Center, Arizona State University, Tempe, AZ, USA

[+]These authors contributed equally

*Corresponding authors

Email: Meg.Bruening@asu.edu; cwhisner@asu.edu

Page S-4, Table S1. Alpha and beta diversity metrics for food insecure and food secure status.

Pages S-5 to S-6, Table S2. Top 10 lowest (set 1) and highest (set 2) ranked genera associated with food security as produced by Songbird analysis.

Page S-7, Table S3. Top 10 lowest (set 1) and highest (set 2) ranked predicted microbial functions associated with food security as produced by Songbird analysis.

Pages S-8 to S-9, Table S4. Taxa generated from gut microbiome analysis with greatest fecal metabolite cooccurrence as produced by mmvec analysis.

Page S-10, Figure S1. Pattern search analysis performed via SparCC identified four dominant taxa, **a** *Bacteroides*, **b** *Blautia*, **c** *Alistipes*, and **d** *Faecalibacterium*. The top 25 taxa are displayed, ranked by correlation coefficient with red and blue denoting positive and negative correlations, respectively. The adjacent heat map for each plot displays which group the relative abundance is weighted towards (red = increased; blue = decreased).

Page S-11, Figure S2. Predicted functional metabolic pathways by food security status. **a** Comparison of the log ratio of the 10 lowest ("Set 1") and 10 highest ("Set 2") ranked pathways associated with food security status (after filtering, n = 58). **b** Food secure status had a significantly greater log ratio of Set 2 compared to Set 1 (Mann-Whitney U test: $p < 0.05$).

Page S-12, Figure S3. Feature-wise distribution of metabolite concentrations before and after normalization. Data were square root transformed and auto scaled to approximate normality.

Page S-13, Figure S4. RF analysis performed on metabolite data using 500 decision trees indicates five outlying subjects between groups.

Page S-14, Figure S5. Two-dimensional scores plot of PCA conducted using the entire set of captured metabolites between groups. PCA was performed using normalized metabolite concentrations.

Page S-15, Figure S6. Disease and enzyme enrichment analysis performed via LASSO regression. **a** Disease enrichment analysis performed using 44 metabolite sets reported in human feces. **b** Enzyme enrichment analysis performed using 912 metabolite sets predicted to change in the case of dysfunctional enzymes.

Page S-16, Figure S7. Pathway analysis performed a global test of relative-betweenness centrality. Detected study metabolites were mapped to the KEGG human database.

Page S-17, Figure S8. Quality-based filtering processes for microbiome data. Quality scores based on rarefaction assessment for **a** Forward and **b** Reverse reads. **c** Low abundance/low

prevalence amplicon sequence variants (ASVs) were filtered out using the feature-table plugin with the filter-samples method (--p-min-frequency 10; --p-min-samples 2). **d** To account for uneven sequencing depth between samples, normalization was performed via alpha rarefaction for observed features and Shannon index. Based on the ASV feature table, a p-min-depth of 10 and a p-max-depth of 120,000 was used. **e** Based on assessment of alpha rarefaction a threshold of 22,000 sequences/sample was established leaving 58/60 high quality samples for analysis (participants DW09 and DW96 were removed).

Table S1. Alpha and beta diversity metrics for food insecure and food secure status.

**Alpha diversity metric**	**Food insecure (*n* = 22)**	**Food secure (*n* = 38)**	***F*-statistic**	***p***
Observed Features	161.50 ± 10.89	143.08 ± 7.14	2.21	0.143
Faith's PD	16.77 ± 0.93	15.33 ± 0.77	1.41	0.241
Pielou's E	0.71 ± 0.02	0.68 ± 0.01	3.96	0.050
Shannon	5.21 ± 0.16	4.85 ± 0.13	4.13	0.047
**Beta diversity metric**	**Sum of Squares**	***F*-statistic**	R^2	***p***
Jaccard	0.374	1.252	0.02	0.050
Bray Curtis	0.248	0.922	0.01	0.579
Unweighted UniFrac	0.224	1.376	0.02	0.080
Weighted UniFrac	0.084	1.069	0.02	0.326

Alpha diversity values for food security status displayed as mean ± SD.

Table S2. Top 10 lowest (set 1) and highest (set 2) ranked genera associated with food security as produced by Songbird analysis.

Set 1	Feature	Intercept	Log FC (Food Insecure)
1	d__Bacteria;p__Proteobacteria;c__Gammaproteobacteria; o__Enterobacterales;f__Enterobacteriaceae;__	0.813	-2.621
2	d__Bacteria;p__Firmicutes;c__Clostridia;o__Lachnospiral es;f__Lachnospiraceae;g__Eisenbergiella	2.650	-2.116
3	d__Bacteria;p__Firmicutes;c__Clostridia;o__Oscillospirale s;f__Ruminococcaceae;g__Angelakisella	-0.973	-1.643
4	d__Bacteria;p__Bacteroidota;c__Bacteroidia;o__Bacteroid ales;f__Porphyromonadaceae;g__Porphyromonas	0.782	-1.473
5	d__Bacteria;p__Firmicutes;c__Clostridia;o__Lachnospiral es;f__Lachnospiraceae;g__Lachnospiraceae_UCG-001	0.303	-1.454
6	d__Bacteria;p__Firmicutes;c__Clostridia;o__Lachnospiral es;f__Defluviitaleaceae;g__Defluviitaleaceae_UCG-011	0.818	-1.432
7	d__Bacteria;p__Firmicutes;c__Negativicutes;o__Veillonell ales-Selenomonadales;f__Veillonellaceae;g__Veillonella	-0.537	-1.417
8	d__Bacteria;p__Firmicutes;c__Clostridia;o__Oscillospirale s;f__Ruminococcaceae;g__[Eubacterium]_siraeum_group	4.311	-1.317
9	d__Bacteria;p__Firmicutes;c__Clostridia;o__Clostridia_va dinBB60_group;f__Clostridia_vadinBB60_group;g__Clostri dia_vadinBB60_group	1.323	-1.314
10	d__Bacteria;p__Firmicutes;c__Bacilli;o__Erysipelotrichale s;f__Erysipelotrichaceae;g__Turicibacter	-0.537	-1.258

Set 2	Feature	Intercept	Log FC (Food secure)
1	d__Bacteria;p__Firmicutes;c__Clostridia;__;__;__	1.538	2.830
2	d__Bacteria;p__Firmicutes;c__Negativicutes;o__Veillonell ales- Selenomonadales;f__Veillonellaceae;g__Megasphaera	-4.670	2.507
3	d__Bacteria;p__Firmicutes;c__Bacilli;o__Erysipelotrichale s;f__Erysipelotrichaceae;g__Holdemanella	-1.853	2.350
4	d__Bacteria;p__Firmicutes;c__Bacilli;o__RF39;f__RF39;g __RF39	-5.227	1.399
5	d__Bacteria;p__Proteobacteria;c__Gammaproteobacteria; o__Burkholderiales;f__Sutterellaceae;g__Sutterella	2.359	1.347
6	d__Bacteria;p__Firmicutes;c__Clostridia;o__Oscillospirale s;f__Ruminococcaceae;__	-0.702	1.317
7	d__Bacteria;p__Bacteroidota;c__Bacteroidia;o__Bacteroid ales;f__Prevotellaceae;g__Prevotella	0.058	1.194
8	d__Bacteria;p__Firmicutes;c__Clostridia;o__Oscillospirale s;f__Oscillospiraceae;__	-2.858	1.092

9	d__Bacteria;p__Firmicutes;c__Bacilli;o__Erysipelotrichales;f__Erysipelatoclostridiaceae;g__Erysipelatoclostridium	-0.513	1.034
10	d__Bacteria;p__Firmicutes;c__Clostridia;o__Clostridia;f__Hungateiclostridiaceae;g__Ruminiclostridium	2.917	0.926

Table S3. Top 10 lowest (set 1) and highest (set 2) ranked predicted microbial functions associated with food security as produced by Songbird analysis.

Set 1	Feature	Intercept	Log FC (Food Insecure)
1	Adenosylcobinamide hydrolase	-3.527	-2.089
2	GDP-perosamine synthase	4.581	-1.881
3	Chlorite O(2)-lyase	-2.575	-1.741
4	"1,3-propanediol dehydrogenase"	1.543	-1.714
5	IgA-specific serine endopeptidase	-2.857	-1.484
6	3-deoxy-D-manno-octulosonic acid kinase	-3.027	-1.399
7	Nitric-oxide reductase (cytochrome c)	-3.597	-1.353
8	Ribonuclease T(2)	-4.853	-1.346
9	"Guanosine-3',5'-bis(diphosphate) 3'-diphosphatase"	-4.193	-1.307
10	Nitrite reductase (NO-forming)	-4.015	-1.302
**Set 2**	**Feature**	**Intercept**	**Log FC (Food secure)**
1	UDP-N-acetylmuramoylpentapeptide-lysine N(6)-alanyltransferase	-4.196	2.054
2	Phosphonoacetate hydrolase	1.728	1.672
3	Arginyltransferase	-4.138	1.625
4	Quinate/shikimate dehydrogenase	0.667	1.421
5	Acylaminoacyl-peptidase	-3.333	1.302
6	4-hydroxybenzoyl-CoA thioesterase	0.859	1.256
7	Allantoin racemase	-0.322	1.246
8	6-phospho-beta-galactosidase	-4.226	1.224
9	Site-specific DNA-methyltransferase (cytosine-N(4)-specific)	-0.662	1.202
10	Acetolactate decarboxylase	-1.117	1.151

Table S4. Taxa generated from gut microbiome analysis with greatest fecal metabolite cooccurrence as produced by mmvec analysis.

Taxa	Metabolite	mmvecRank
**Food Insecure**		
d__Bacteria;p__Firmicutes;c__Bacilli;o__Erysipelotrichales;f__Erysipelotrichaceae;g__[Clostridium]_innocuum_group	L_Alloisoleucine_Leucine_Norleucine	4.518
	Isoleucine	4.173
	Valine	3.703
	Phenylalanine	3.423
	Proline	3.049
d__Bacteria;p__Firmicutes;c__Clostridia;o__Lachnospirales;f__Lachnospiraceae;g__Lachnospiraceae_FCS020_group	L_Alloisoleucine_Leucine_Norleucine	4.241
	Isoleucine	3.714
	Valine	3.436
	Phenylalanine	3.185
	Isobutyric_acid	2.828
d__Bacteria;p__Firmicutes;c__Clostridia;o__Lachnospirales;f__Lachnospiraceae;g__Lachnospiraceae_UCG-008	L_Alloisoleucine_Leucine_Norleucine	4.638
	Isoleucine	4.449
	Valine	3.795
	Phenylalanine	3.504
	Proline	3.318
d__Bacteria;p__Firmicutes;c__Clostridia;o__Oscillospirales;f__Oscillospiraceae;__	Isoleucine	5.093
	L_Alloisoleucine_Leucine_Norleucine	5.016
	Valine	4.228
	Proline	4.071
	Phenylalanine	3.827
d__Bacteria;p__Firmicutes;c__Clostridia;o__Oscillospirales;f__Ruminococcaceae;__	L_Alloisoleucine_Leucine_Norleucine	4.164
	Isoleucine	3.515
	Valine	3.348
	Phenylalanine	3.140
	Isobutyric_acid	2.989
**Food Secure**		
d__Bacteria;p__Cyanobacteria;c__Cyanobacteriia;o__Chloroplast;f__Chloroplast;g__Chloroplast	Isoleucine	5.088
	L_Alloisoleucine_Leucine_Norleucine	4.647
	Valine	3.585
	Phenylalanine	3.129
	Stearic_acid	2.761
d__Bacteria;p__Firmicutes;c__Bacilli;o__Erysipelotrichales;f__Erysipelotrichaceae;g__[Clostridium]_innocuum_group	Isoleucine	5.155
	L_Alloisoleucine_Leucine_Norleucine	4.775
	Valine	3.687
	Phenylalanine	3.248
	Pyroglutamic_acid	2.673
d__Bacteria;p__Firmicutes;c__Clostridia;o__Lachnospirales;f__Lachnospiraceae;g__GCA-900066575	Creatine	4.884
	Isoleucine	4.104
	Acetylcholine	3.721
	L_Alloisoleucine_Leucine_Norleucine	3.711
	Carnosine	2.951
d__Bacteria;p__Firmicutes;c__Clostridia;o__Lachnospirales;f__Lachnospir	Isoleucine	5.181
	L_Alloisoleucine_Leucine_Norleucine	4.834

aceae;g__Lachnospiraceae_FCS020_group	Valine	3.727
	Phenylalanine	3.291
	Pyroglutamic_acid	2.707
d__Bacteria;p__Firmicutes;c__Clostridia;o__Lachnospirales;f__Lachnospiraceae;g__Lachnospiraceae_UCG-008	Isoleucine	4.652
	L_Alloisoleucine_Leucine_Norleucine	4.265
	Valine	3.068
	Creatine	3.043
	Phenylalanine	2.637

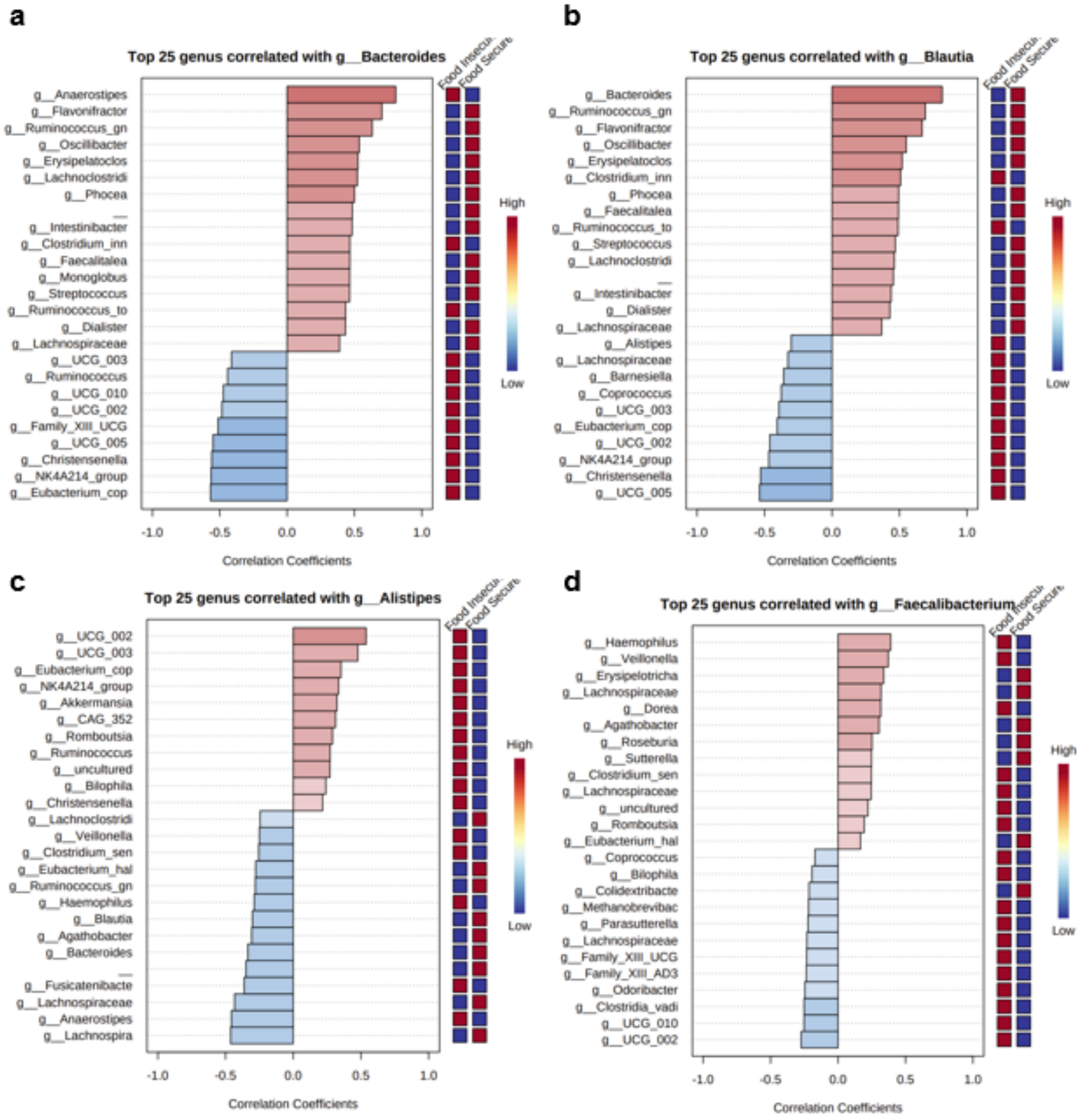

Figure S1. Pattern search analysis performed via SparCC identified four dominant taxa, **a** *Bacteroides*, **b** *Blautia*, **c** *Alistipes*, and **d** *Faecalibacterium*. The top 25 taxa are displayed, ranked by correlation coefficient with red and blue denoting positive and negative correlations, respectively. The adjacent heat map for each plot displays which group the relative abundance is weighted towards (red = increased; blue = decreased).

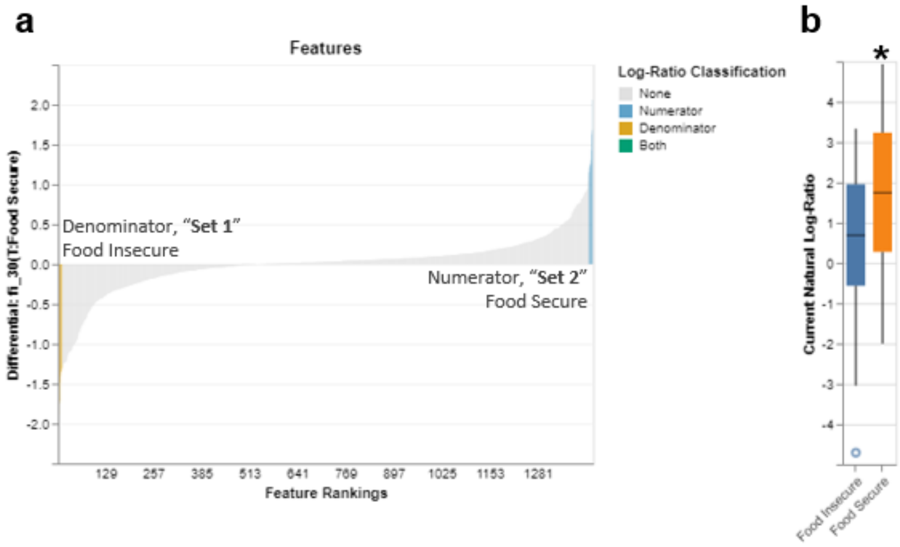

Figure S2. Predicted functional metabolic pathways by food security status. **a** Comparison of the log ratio of the 10 lowest (“Set 1”) and 10 highest (“Set 2”) ranked pathways associated with food security status (after filtering, n = 58). **b** Food secure status had a significantly greater log ratio of Set 2 compared to Set 1 (Mann-Whitney U test: $p < 0.05$).

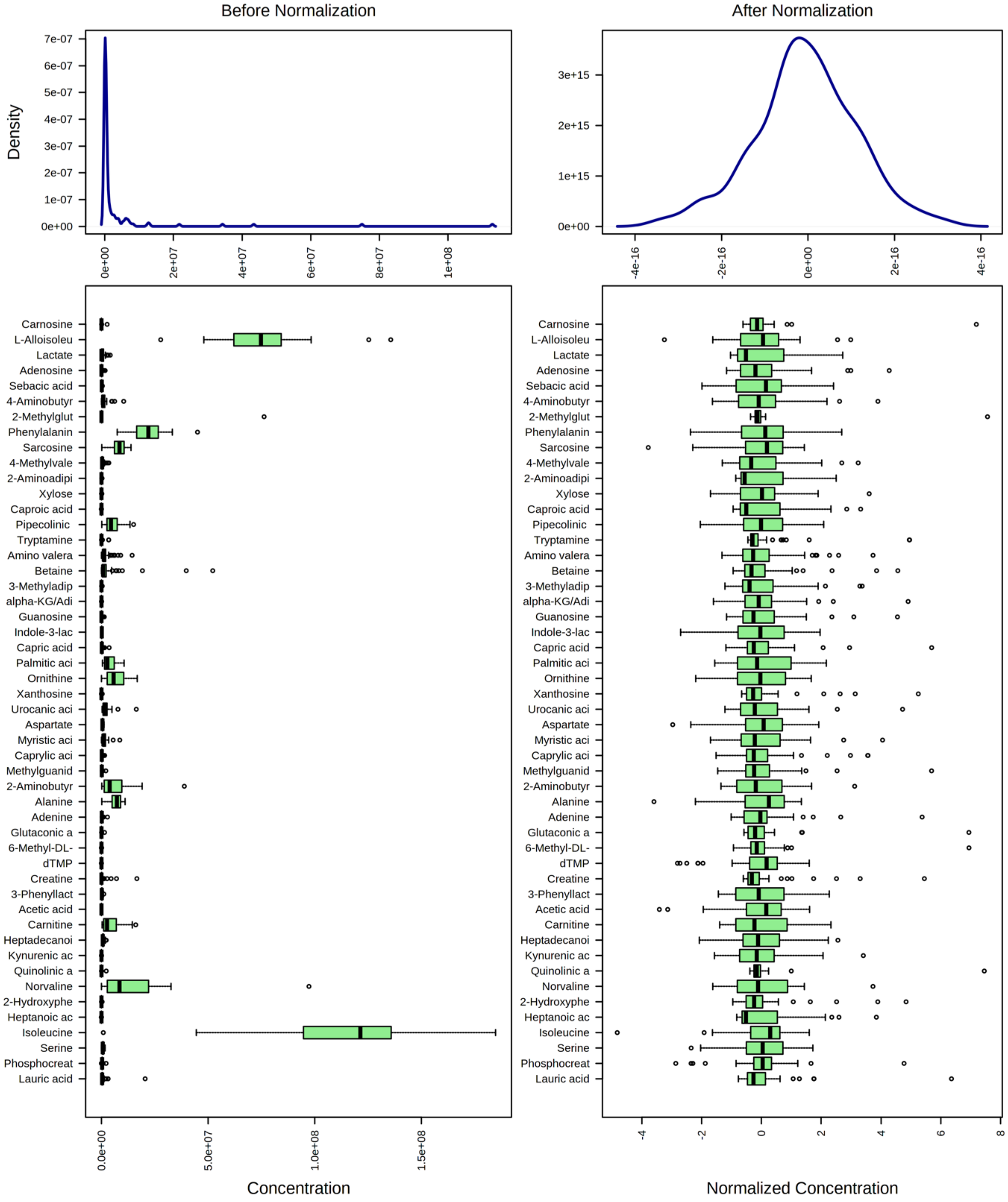

Figure S3. Feature-wise distribution of metabolite concentrations before and after normalization. Data were square root transformed and auto scaled to approximate normality.

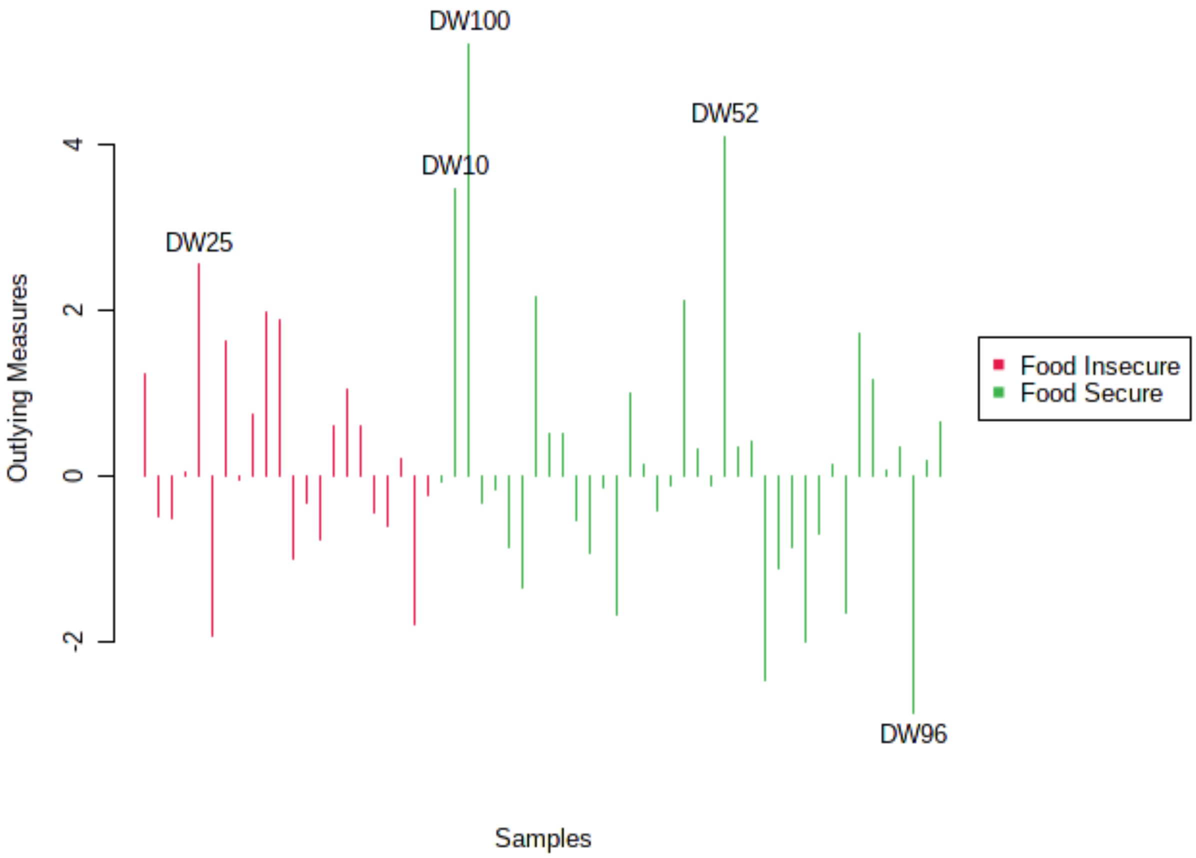

Figure S4. RF analysis performed on metabolite data using 500 decision trees indicates five outlying subjects between groups.

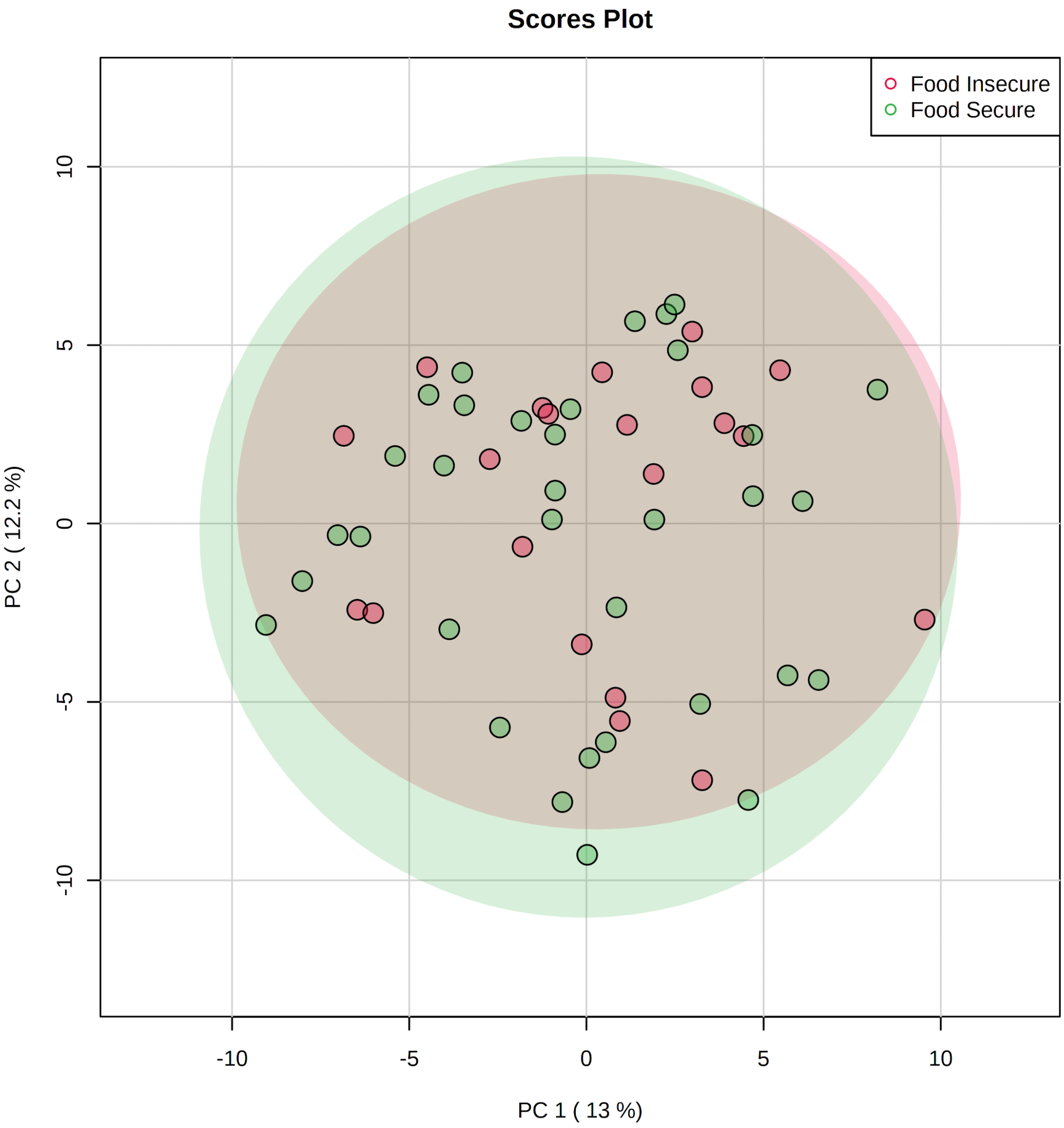

Figure S5. Two-dimensional scores plot of PCA conducted using the entire set of captured metabolites between groups. PCA was performed using normalized metabolite concentrations.

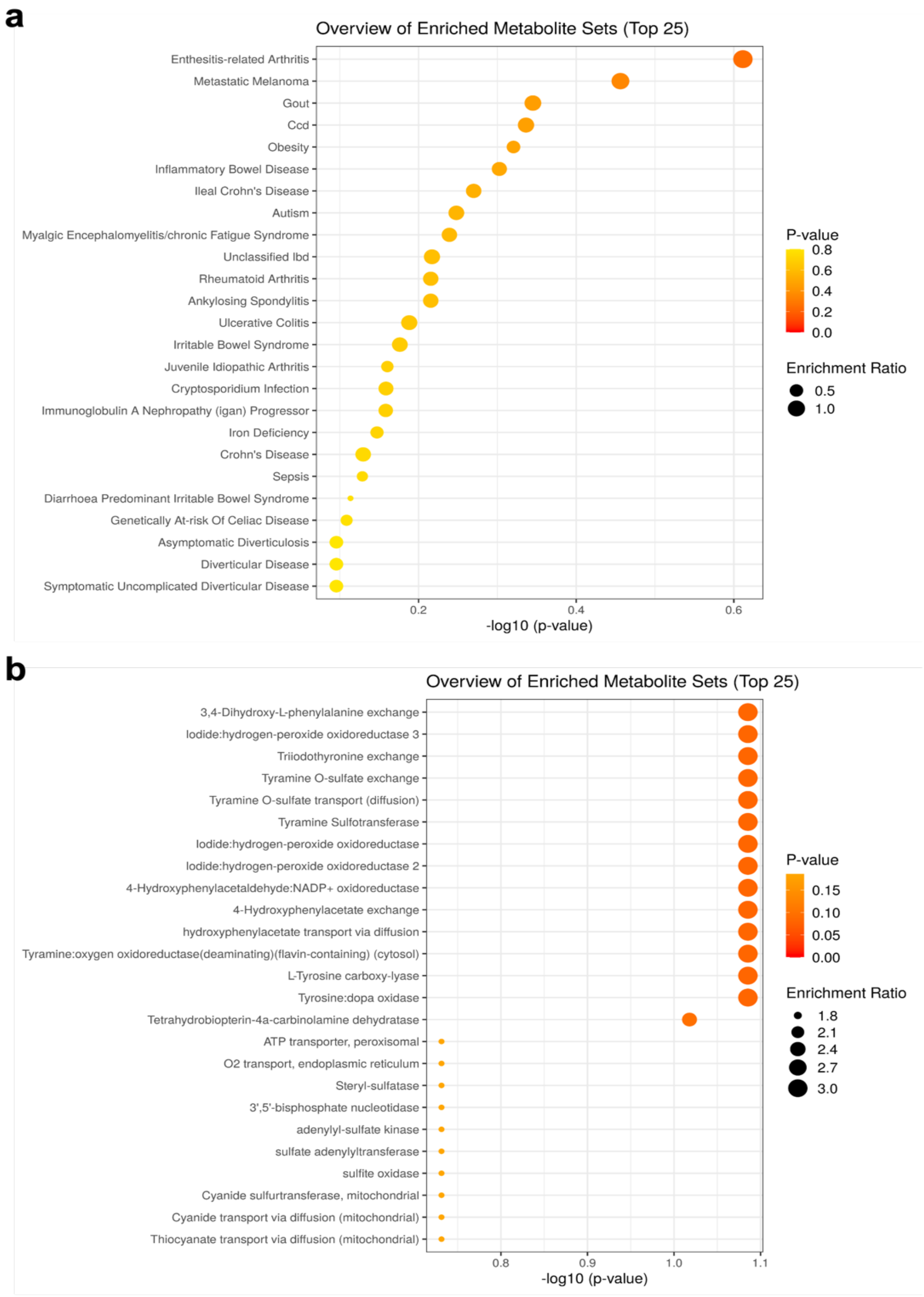

Figure S6. Disease and enzyme enrichment analysis performed via LASSO regression. **a** Disease enrichment analysis performed using 44 metabolite sets reported in human feces. **b** Enzyme enrichment analysis performed using 912 metabolite sets predicted to change in the case of dysfunctional enzymes.

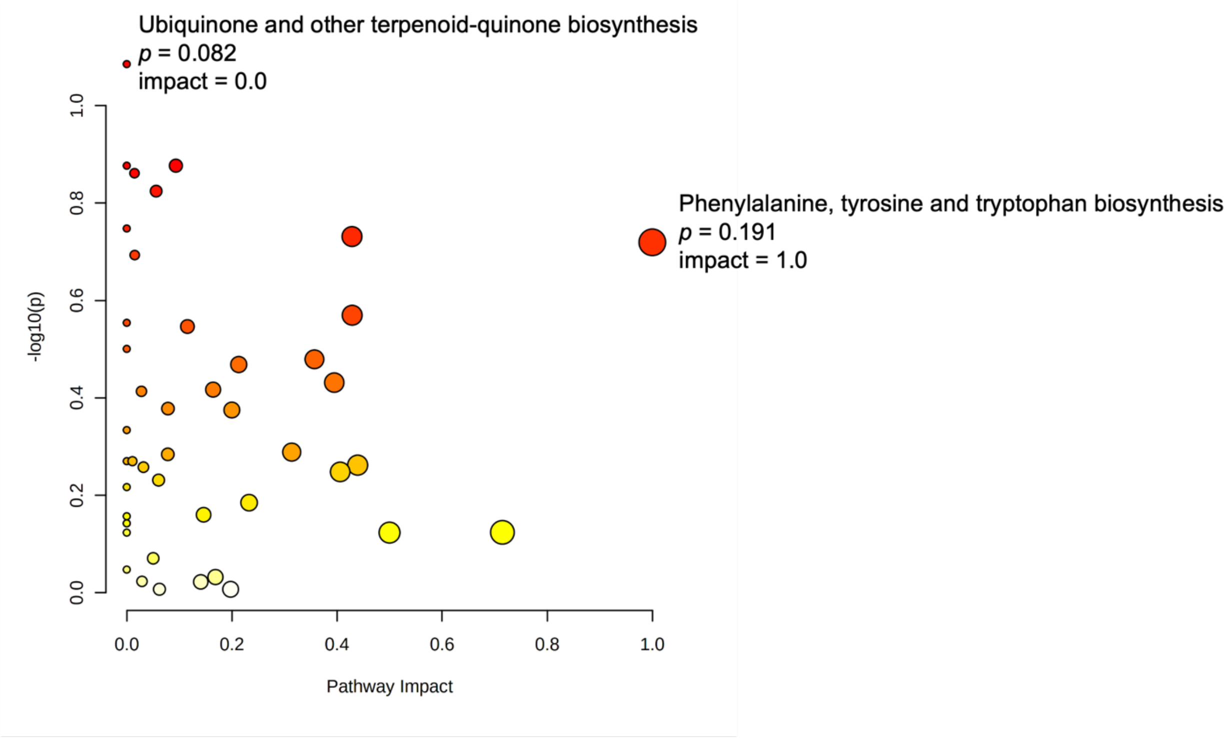

Figure S7. Pathway analysis performed a global test of relative-betweenness centrality. Detected study metabolites were mapped to the KEGG human database.

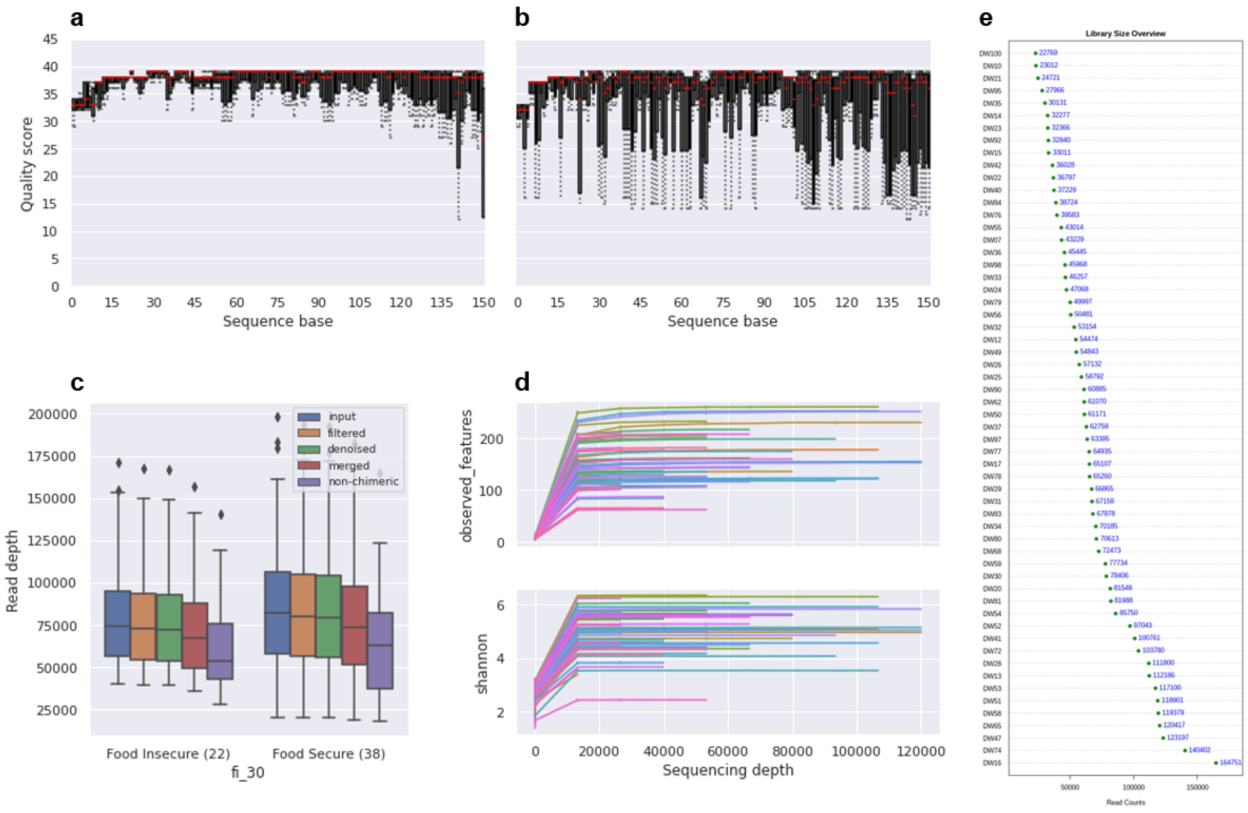

Figure S8. Quality-based filtering processes for microbiome data. Quality scores based on rarefaction assessment for **a** Forward and **b** Reverse reads. **c** Low abundance/low prevalence amplicon sequence variants (ASVs) were filtered out using the feature-table plugin with the filter-samples method (--p-min-frequency 10; --p-min-samples 2). **d** To account for uneven sequencing depth between samples, normalization was performed via alpha rarefaction for observed features and Shannon index. Based on the ASV feature table, a p-min-depth of 10 and a p-max-depth of 120,000 was used. **e** Based on assessment of alpha rarefaction a threshold of 22,000 sequences/sample was established leaving 58/60 high quality samples for analysis (participants DW09 and DW96 were removed).
